# Supplementary material for: Randomized controlled trial of molnupiravir SARS-CoV-2 viral and antibody response in at-risk adult outpatients
Source: Nat Commun. 2024 Feb 23;15:1652. doi: 10.1038/s41467-024-45641-0 (PMC10891158; doi:10.1038/s41467-024-45641-0)
Supplement: Supplementary file 1 — Supplementary Information [file 41467_2024_45641_MOESM1_ESM.pdf]

Supplementary Material: Randomized controlled trial of molnupiravir SARS-CoV-2 viral and antibody response in at-risk adult outpatients

Supplementary Figure 1a | Basic goodness of fit for the viral dynamic model. DV, dependent variable (viral load). Population predictions are the typical predicted for each DV. Individual predictions use the empirical Bayes estimates of the parameters. NPDE: Normalised Prediction Distribution Errors, which are a type of weighted residual and should follow N(0,1). Purple points represent samples above the LLOQ, black dots below. Samples from individual participants are joined and a red smooth is added to compare with either the line of unity (top row) or zero line (bottom row).

a

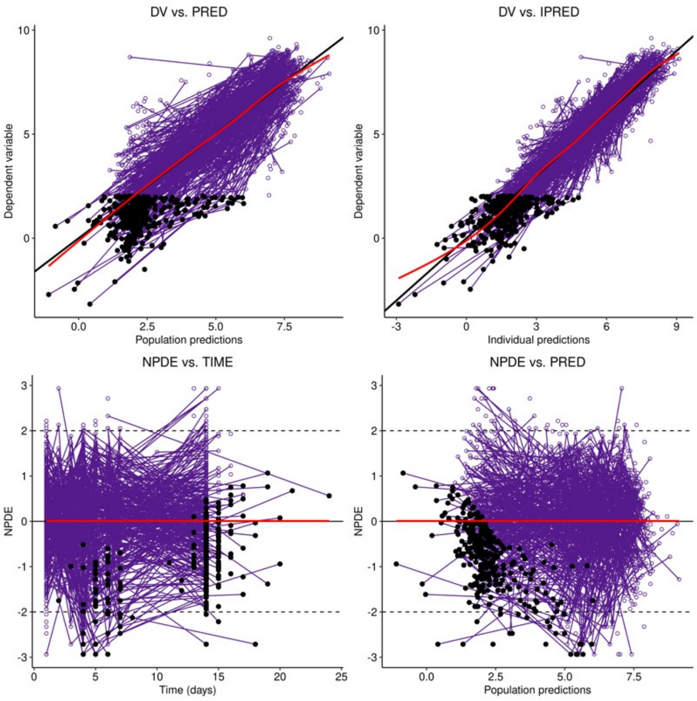

Supplementary Figure 1b | Basic goodness of fit for the antibody dynamic model. DV, dependent variable (spike antibody). Population predictions are the typical predicted for each DV. Individual predictions use the empirical Bayes estimates of the parameters. NPDE: Normalised Prediction Distribution Errors, which are a type of weighted residual and should follow N(0,1). Purple points represent samples from each participant. Samples from individual participants are joined and a red smooth is added to compare with either the line of unity (top row) or zero line (bottom row).

b

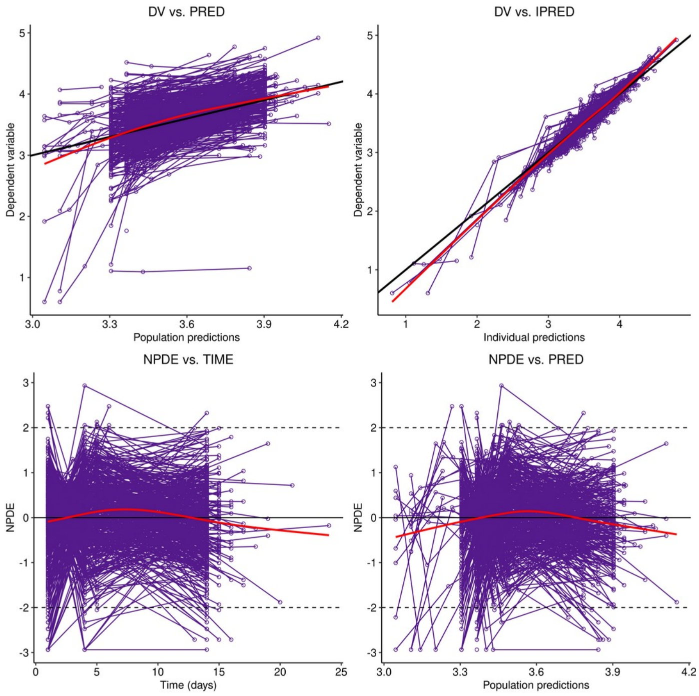

Supplementary Figure 2a | Individual viral load trajectories (open circles) for Usual Care Participants in the intensive sampling arm. Blue line represents population model prediction, red line represents the individual model prediction. black points are observations below the LLOQ

a

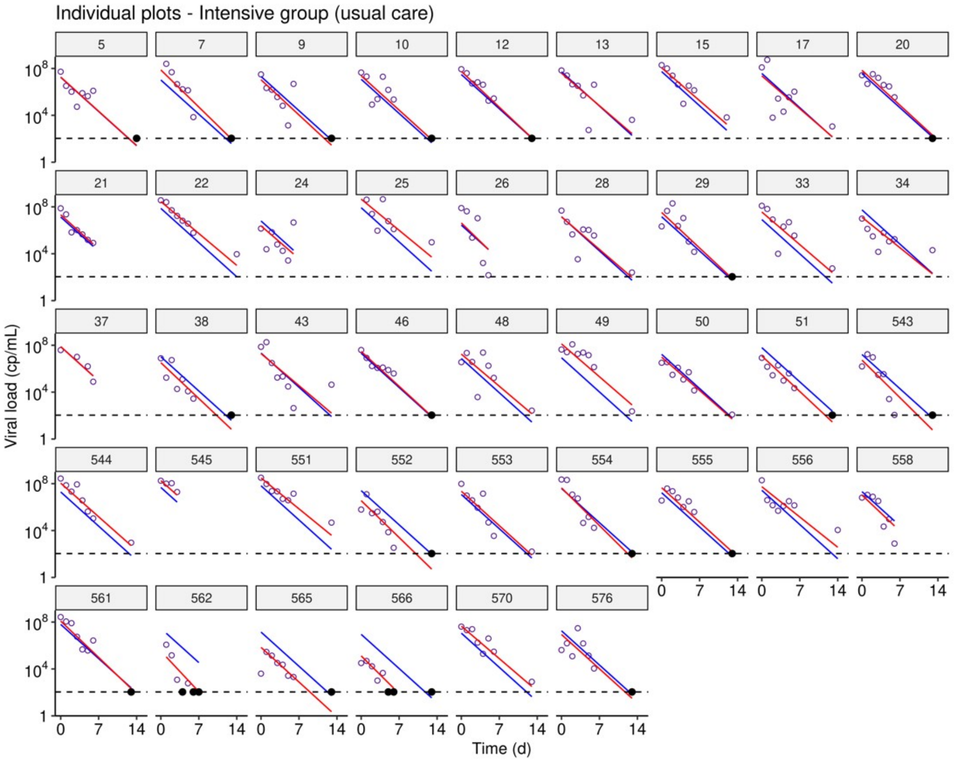

Supplementary Figure 2b | Individual viral load trajectories (open circles) for molnupiravir-treated participants in the intensive sampling arm. Blue line represents population model prediction, red line represents the individual model prediction. Black points are observations below the LLOQ

b

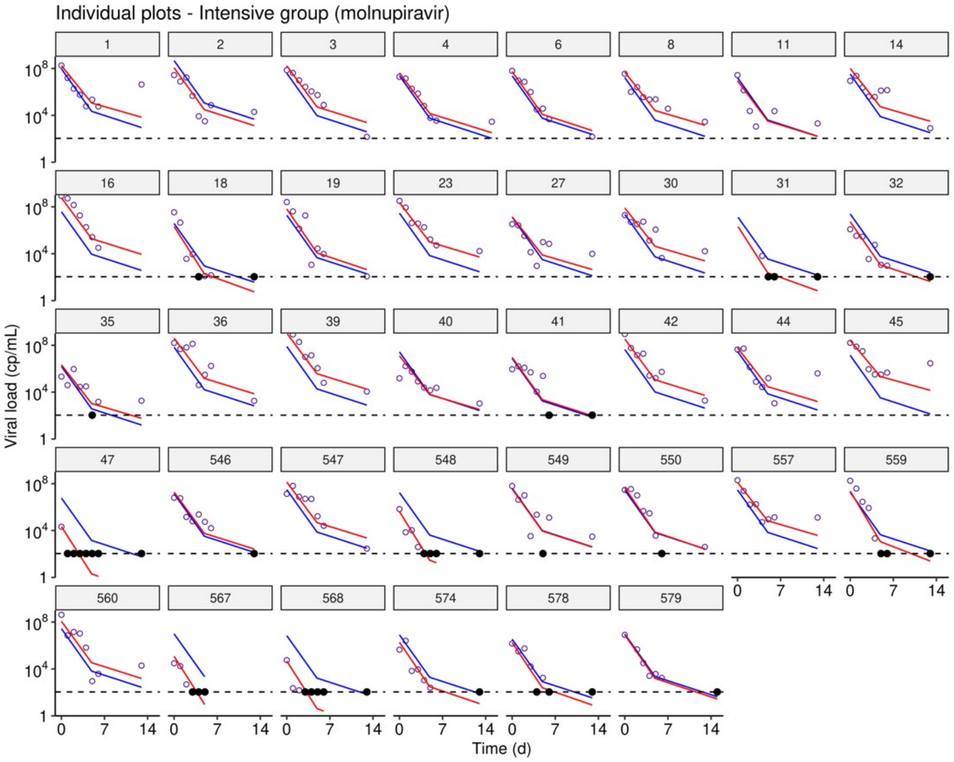

Supplementary Figure 3 | Capillary CRP dynamics with time in molnupiravir treated (purple) and Usual Care (grey). Lines are geometric means, error bars represent 95% CI of the geometric mean.

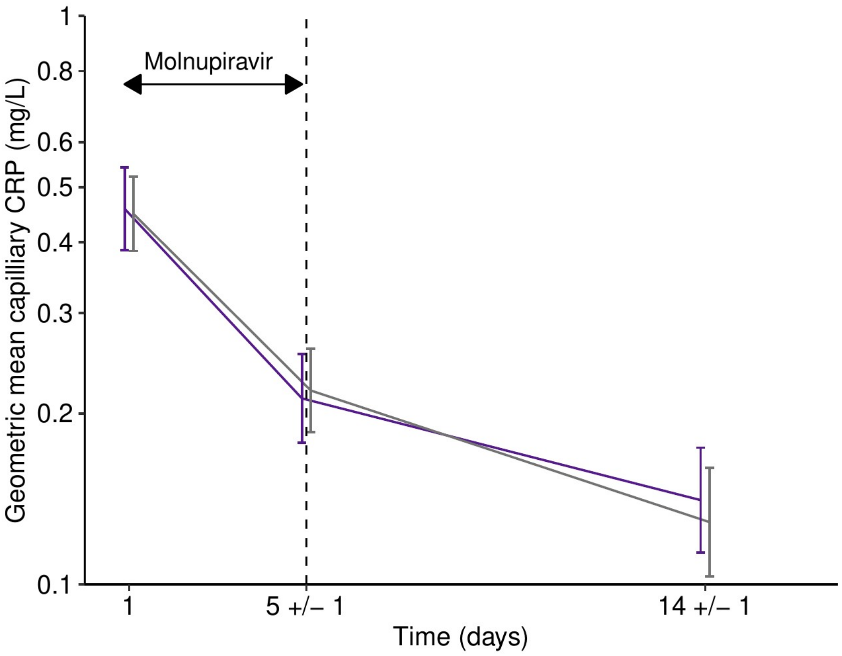

Supplementary Figure 4 | Relationship between SARS-CoV-2 sequencing success and viral load. Box- plot showing the quantitative PCR cycle threshold (CT) values for viral sequences with 90% coverage and mean read depth (MRD) of x10 (n=1437) in grey (PASS) and those (n=137) that failed to reach this threshold in purple (FAIL). Means and standard deviations (boxed area) are shown. All samples failing sequencing had CT values >31. Sequencing of a further 19 samples with CT values of >38 was not attempted. CT values for PASS samples were significantly lower than those for FAIL samples (p<0.001 Mann-Whitney U test).

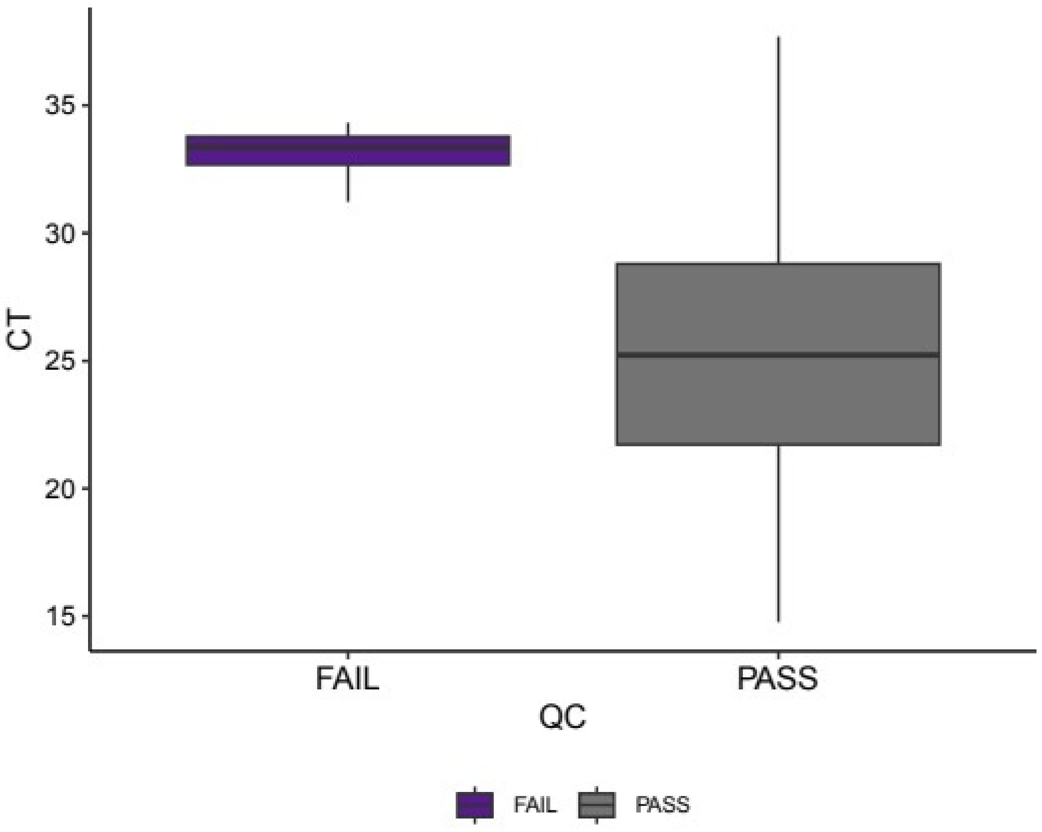

Supplementary Figure 5 | Relationship between number of mutations above 1% single nucleotide allele frequencies detected per sample and the mean read depth of the sample.

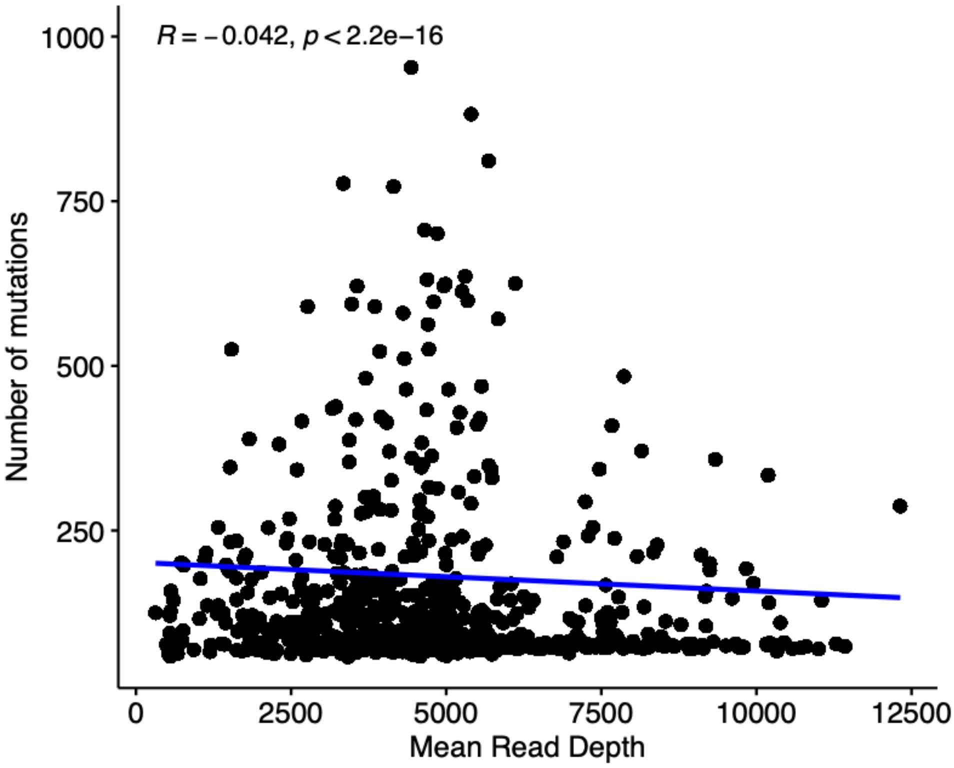

Supplementary Figure 6 | Total transition and transversion mutations over time in viral sequences from molnupiravir and Usual Care participants. Boxplots represent the total number of new single nucleotide polymorphisms (SNPs) above 1% allele frequency detected in samples collected from participants after baseline, divided by treatment group and type of nucleotide substitution. Samples from the molnupiravir treated group are shown in purple, while those from the Usual Care treatment group are shown in grey. Each data point aggregates all post-baseline timepoints for one participant, counting SNPs only once per individual. A total of 831 samples (excluding baseline) were analysed. A Mann-Whitney U test was used (* p<0.05, ** p<0.01, ****p<0.001, **** p<0.0001).

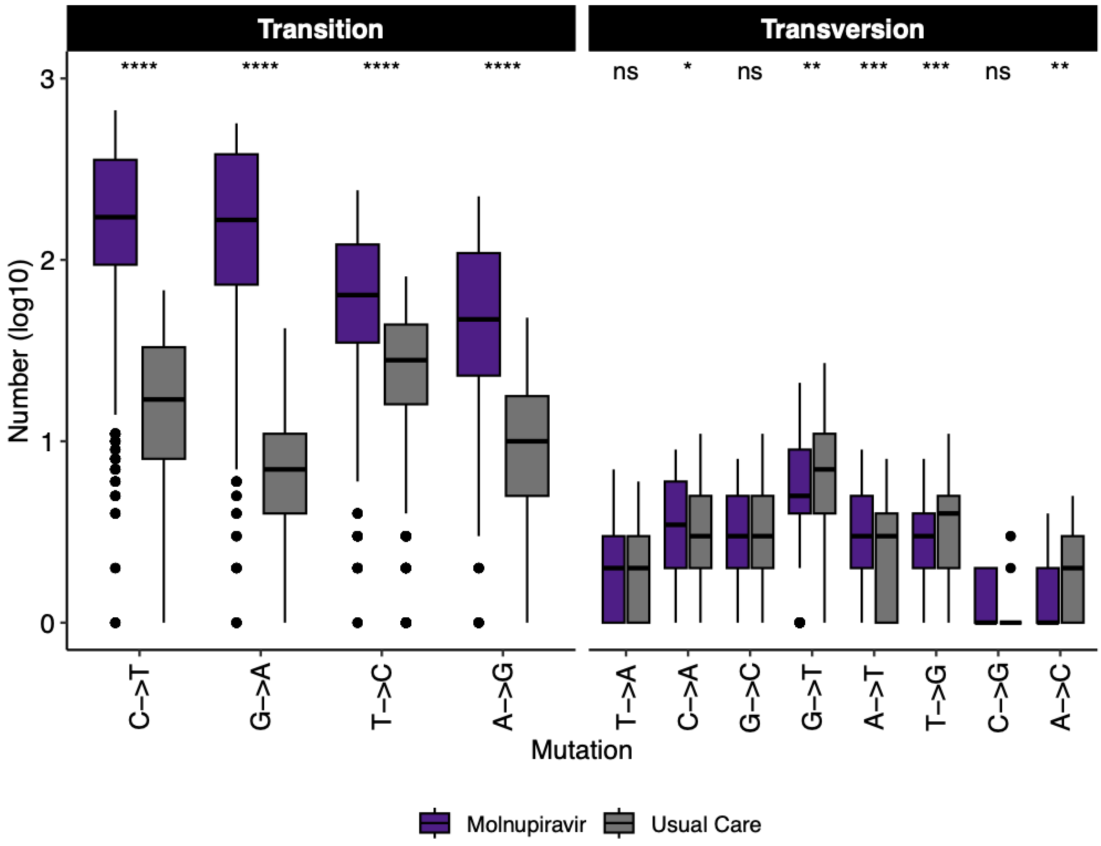

Supplementary Figure 7 | Distribution along the SARS-CoV-2 genome of new post-baseline synonymous and non-synonymous mutations (allele frequency > 5%) at Day 5 (grey) and Day 14 (purple) in molnupiravir and Usual Care participants. The x-axis shows the position along the SARS-CoV-2 genome. The y-axes show the mutation density (mutations at each position/total mutations) for each participant group.

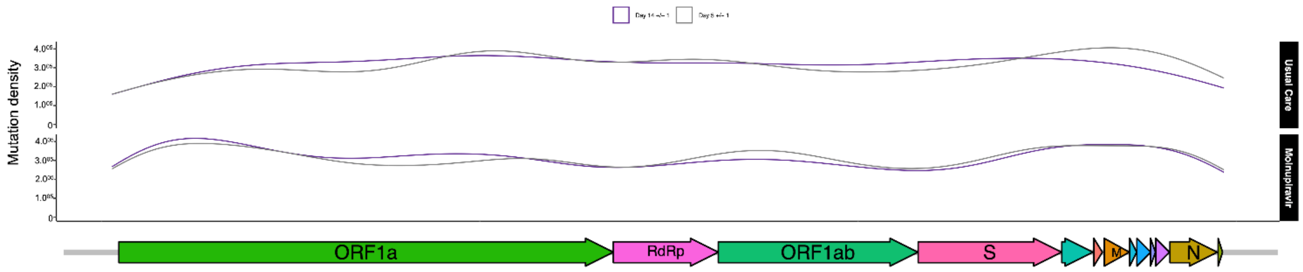

Supplementary Figure 8 | Scatter plot showing the number of new post-baseline mutations above 1% allele frequency (y axis) compared to the transition fraction (number of transitions/total transitions plus transversions) per sample for Days 5 and 14 (x axis). Samples are colour-coded by the treatment received: molnupiravir: purple, and Usual Care: grey. The black dotted line represents the transition fraction threshold below which all Usual Care samples cluster. Day 14 molnupiravir-treated samples which cluster with Day 14 Usual Care samples are shown in red.

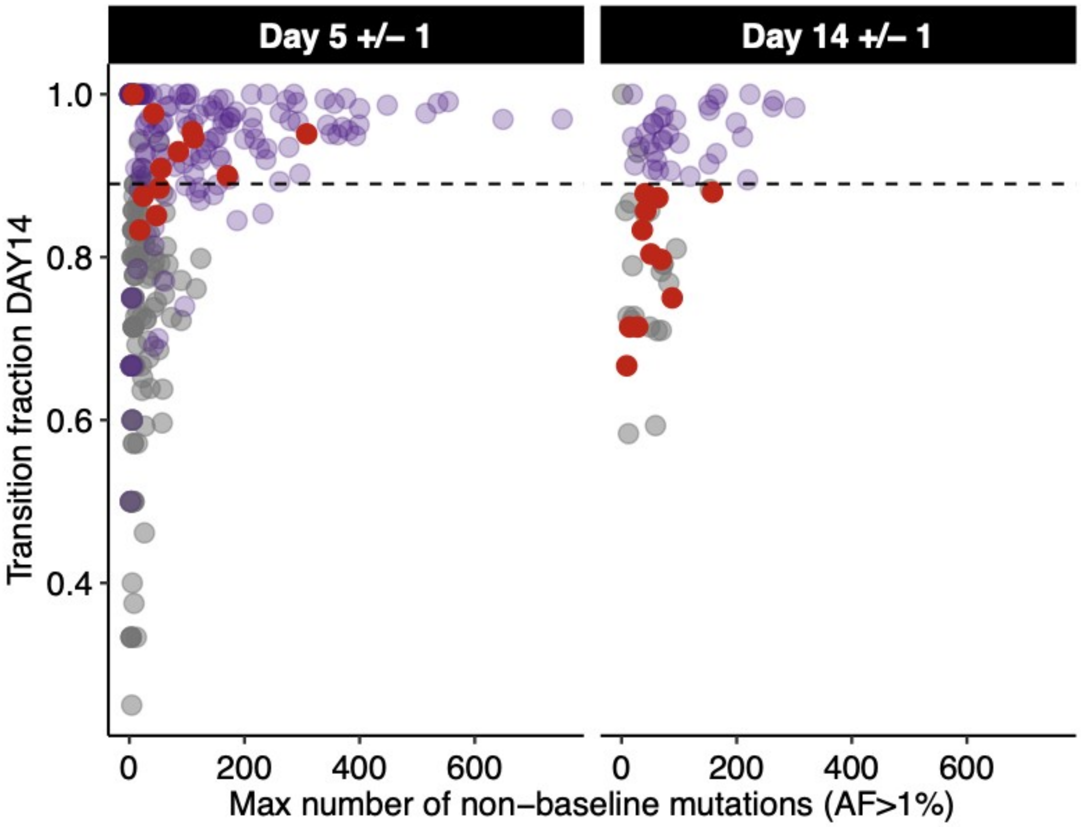

Supplementary Figure 9 | Numbers and frequency of participants with new post-baseline spike escape or molnupiravir resistance mutations at >1% frequency during treatment (Days 2-5) or at Day 14. Participants are divided by treatment (molnupiravir or Usual Care). 'Resolved' participants are those that do not have detectable viral load at Day 14, and 'persistent' participants are those with detectable viral load above LLOQ at ≥ Day 14. Where a participant has more than one sample taken between Days 2-5, the mean number of relevant SNPs detected/participant is included. The size of the circle indicates the number of participants in that group with one or more relevant new mutations by mutation-category and time point. The heat map shows the proportion of participants in each group with a new relevant mutation by mutation-category and time point.

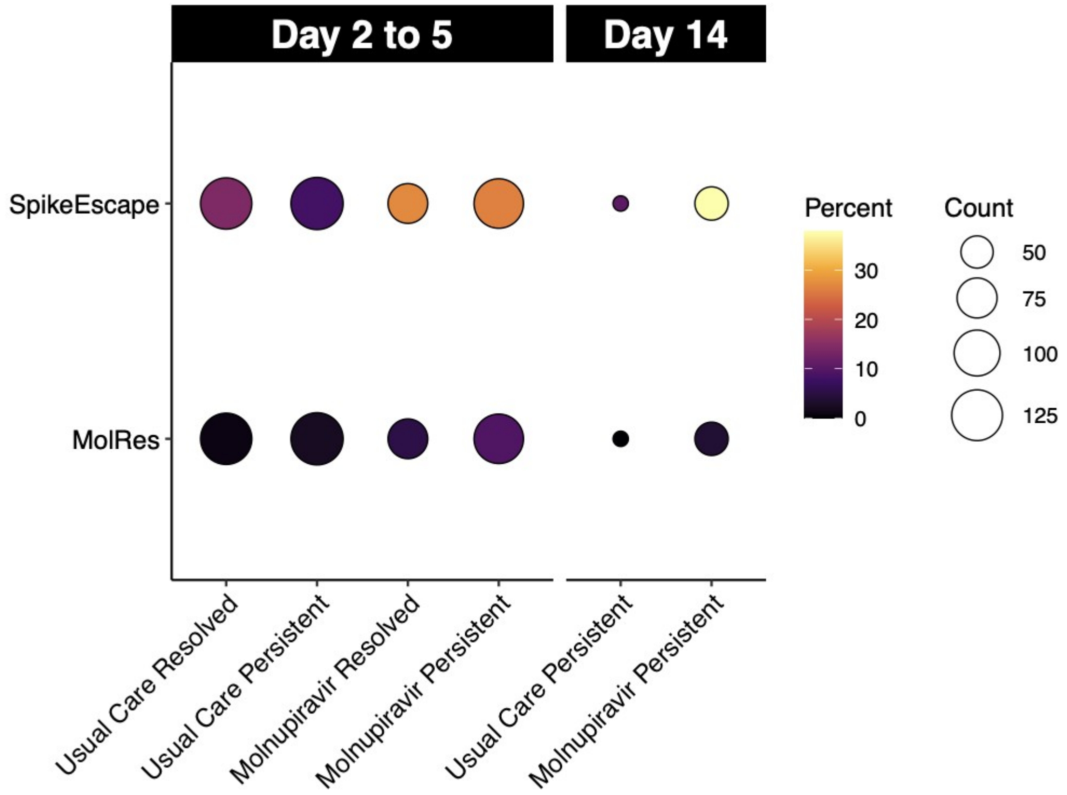

Supplementary Figure 10 | Viral load trajectories (mean and std deviation) for participants with any ORF1a post-baseline missense mutation at any time point which was above the consensus level (allele frequency (AF) >50%) in red, below the consensus level (1-50%) in purple or with no mutations (grey), for molnupiravir-treated and Usual Care groups. Participants included (441) were required to have viral load data for baseline, Day 5 and Day 14 and sequence data for all positive viral loads.

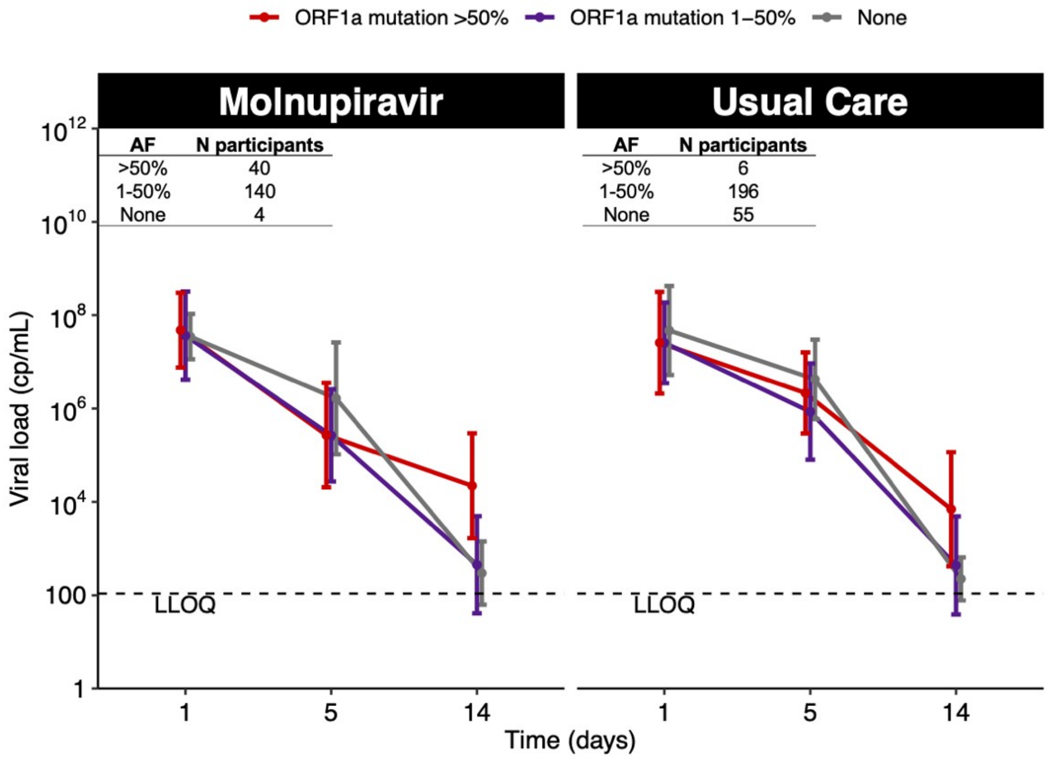

Supplementary Figure 11 | Structure of the SARS-CoV-2 RNA-dependent RNA polymerase based on PDB:6M71 (https://www.rcsb.org/structure/6m71). The catalytic site of the protein is highlighted in orange with the recurrent mutation found in our study at position 716 highlighted in purple.

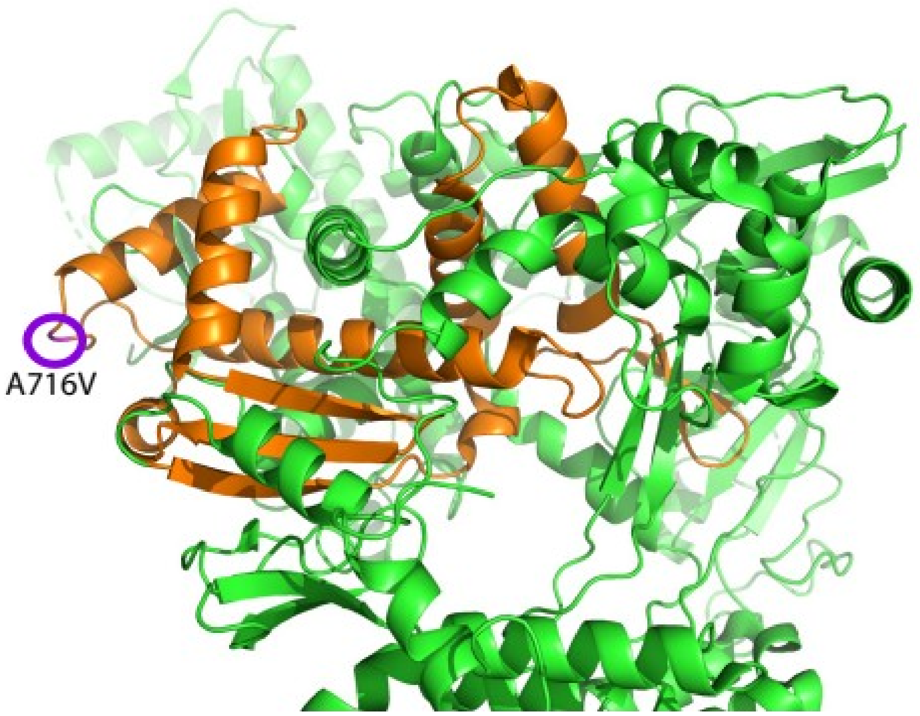

Supplementary Figure 12 | Viral culture results. Plots demonstrating the difference between swab samples from Usual Care (n=558) or molnupiravir-treated (n=425) samples tested for viable virus culture in Calu-3 cells. Samples were characterised as culture positive (filled bar) requiring both observed cytopathogenic effects and a positive lateral flow immunochromatography test of culture medium, and otherwise were designated culture negative (empty bar). a, The participant's viral load at time of sample as determined by PCR (median ± 95% CI). b, The number of transport days between the swab collection and arrival at laboratory (geometric mean ± 95% CI). c, The number of days-post symptom onset at time of sample collection. d, Supernatant from a subset of Calu-3 cultured samples were tested by qRT-PCR using primers to SARS-CoV-2 N gene (n= 31) (geometric mean ± 95% CI). One-way ANOVA was used to calculate significance between specific groups, where **** = p<0.0001, ns = not significant.

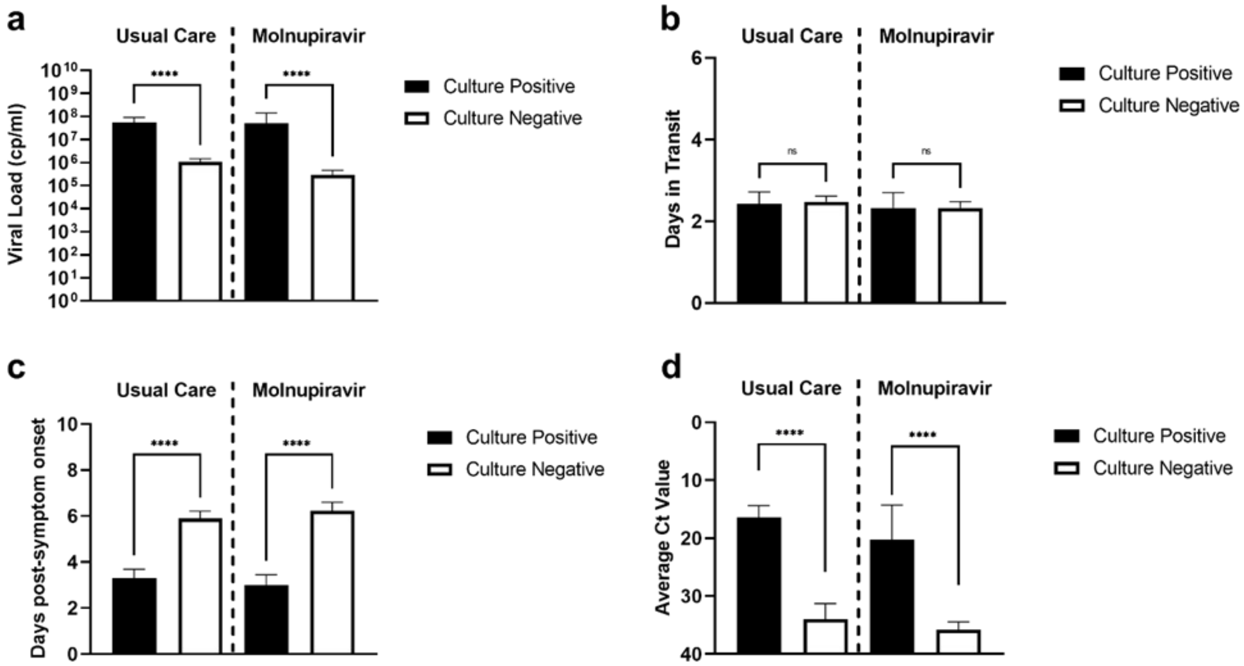

Supplementary Table 1 | Number of participants and collected samples for both less intensive and intensive cohorts for baseline (Day 1), Day 5 and Day 14. The numbers (frequency) successfully sequenced is shown overall and for those with paired baseline and either/both Days 5 and 14 samples.

		**Day 1**		**Day 5**		**Day 14**	
	Treatment	Less intensely sampled	Intensely sampled	Less intensely sampled	Intensely sampled	Less intensely sampled	Intensely sampled
**Total participants**	Molnupiravir	215	38	207	38	171	34
**Total participants sequenced**		212 (98.6%)	37 (97.4%)	124 (59.9%)	32 (84.2%)	33 (19.3%)	13 (34.2%)
**Participants with Paired baseline + at least one other sequence**		130 (60.5%)	31 (81.6%)	124 (59.9%)	31 (81.6%)	33 (19.3%)	13 (34.2%)
**Total participants**	Usual Care	282	42	246	42	208	35
**Total participants sequenced**		277 (98.2%)	40 (95.2%)	175 (71.13%)	41 (97.6%)	17 (8.1%)	4 (11.4%)
**Participants with Paired baseline + at least one other sequence**		174 (61.7%)	40 (95.2%)	171 (69.5%)	40 (95.2%)	17 (8.1%)	4 (11.4%)

Supplementary Table 2 | Lineage assignment of baseline samples (Day 1) from participants for whom viral sequence was available by treatment group.

VOC_VUI	LINEAGE	Number of participants	Percentage	Treatment
**Omicron (BA.2-like)**	BA.2	161	72.85%	Molnupiravir
**Omicron (BA.2-like)**	BA.2.9	11	4.98%	Molnupiravir
**Omicron (BA.2-like)**	BA.2.3	10	4.52%	Molnupiravir
**Omicron (BA.2-like)**	BA.2.1	8	3.62%	Molnupiravir
**Omicron (BA.2-like)**	BA.2.23	8	3.62%	Molnupiravir
**Omicron (BA.2-like)**	BA.2.10	4	1.81%	Molnupiravir
**Omicron (BA.2-like)**	BA.2.18	3	1.36%	Molnupiravir
**Omicron (BA.1-like)**	BA.1.1	2	0.90%	Molnupiravir
**Omicron (BA.2-like)**	BA.2.22	2	0.90%	Molnupiravir
**Omicron (BA.2-like)**	BA.2.37	2	0.90%	Molnupiravir
**Omicron (BA.2-like)**	BA.2.3.9	1	0.45%	Molnupiravir
**Omicron (BA.2-like)**	BA.2.36	1	0.45%	Molnupiravir
**Omicron (BA.2-like)**	BA.2.39	1	0.45%	Molnupiravir
**Omicron (BA.2-like)**	BA.2.50	1	0.45%	Molnupiravir
**Omicron (BA.2-like)**	BA.2.51	1	0.45%	Molnupiravir
**Omicron (BA.2-like)**	BA.2.52	1	0.45%	Molnupiravir
**Omicron (BA.2-like)**	BA.2.8	1	0.45%	Molnupiravir
**Omicron (BA.2-like)**	XN	1	0.45%	Molnupiravir
**Omicron (Unassigned)**	XL	1	0.45%	Molnupiravir
**Omicron (Unassigned)**	XQ	1	0.45%	Molnupiravir
**Omicron (BA.2-like)**	BA.2	203	75.19%	Usual Care
**Omicron (BA.2-like)**	BA.2.9	16	5.93%	Usual Care
**Omicron (BA.2-like)**	BA.2.3	10	3.70%	Usual Care
**Omicron (BA.2-like)**	BA.2.1	6	2.22%	Usual Care
**Omicron (BA.2-like)**	BA.2.23	6	2.22%	Usual Care
**Omicron (BA.2-like)**	BA.2.10	5	1.85%	Usual Care
**Omicron (BA.2-like)**	BA.2.22	3	1.11%	Usual Care
**Omicron (BA.2-like)**	BA.2.18	2	0.74%	Usual Care
**Omicron (BA.2-like)**	BA.2.38	2	0.74%	Usual Care
**Omicron (XE-like)**	XE	2	0.74%	Usual Care
**Omicron (BA.1-like)**	BA.1.1	1	0.37%	Usual Care
**Omicron (BA.1-like)**	BA.1.1.15	1	0.37%	Usual Care
**Omicron (BA.2-like)**	BA.2.10.1	1	0.37%	Usual Care
**Omicron (BA.2-like)**	BA.2.31	1	0.37%	Usual Care
**Omicron (BA.2-like)**	BA.2.41	1	0.37%	Usual Care
**Omicron (BA.2-like)**	BA.2.45	1	0.37%	Usual Care
**Omicron (BA.2-like)**	BA.2.5	1	0.37%	Usual Care
**Omicron (BA.2-like)**	BA.2.50	1	0.37%	Usual Care

**Omicron (BA.2-like)**	BA.2.55	1	0.37%	Usual Care
**Omicron (BA.2-like)**	BA.2.6	1	0.37%	Usual Care
**Omicron (BA.2-like)**	BA.2.65	1	0.37%	Usual Care
**Omicron (BA.2-like)**	BA.2.8	1	0.37%	Usual Care
**Omicron (BA.5-like)**	BF.1	1	0.37%	Usual Care
**Omicron (Unassigned)**	BA.2	1	0.37%	Usual Care
**Omicron (Unassigned)**	XQ	1	0.37%	Usual Care

Supplementary Table 3 | Baseline characteristics of the thirteen molnupiravir-treated group samples with viral sequences at day 14 which appeared to have low mutagenesis (≤89%) comparable to the Usual Care group. Asterisks highlight the four participants who also had Day 5 transition fractions similar to the Usual Care group. nMut refers to the number of mutations detected at different allele frequency thresholds.

Participant ID	Time (Days)	Transition Fraction	nMut (>1%)	nMut (>5%)	nMut (>50%)	CRP (mg/L)	Sex	Lung disease	Heart disease	Kidney disease	Liver disease	Neuro disease	Diabetes	Immune disease
2	4	1.000	7	0	0	0.18	M	Yes	No	Yes	No	No	Yes	Yes
80	4	0.909	55	0	0	0.19	F	Yes	Yes	No	No	No	No	No
98	4	1.000	2	0	0	0.65	F	Yes	No	No	No	No	No	Yes
113*	4	0.885	52	38	0	0.15	F	No	No	No	No	No	No	No
181	4	0.951	308	3	2	0.13	M	No	No	No	No	No	No	No
227	4	0.900	170	3	0	1.03	F	No	No	No	No	No	No	No
246*	4	0.833	18	4	2	0.2	M	No	No	No	No	No	No	No
248	4	0.954	109	2	0	0.07	F	No	No	No	No	No	No	No
286	4	0.976	42	0	0	0.13	F	No	No	No	No	No	No	No
308*	4	0.851	47	17	0	0.18	F	No	No	No	No	No	Yes	No
366*	4	0.875	24	17	0	0.29	F	No	Yes	No	No	No	Yes	No
2	5	0.919	135	61	0	0.18	M	Yes	No	Yes	No	No	Yes	Yes
387	5	0.946	112	1	2	0.18	M	No	Yes	No	No	No	No	No
536	5	0.929	85	7	2	0.6	F	Yes	No	No	No	No	Yes	No
2	6	0.973	73	69	0	0.18	M	Yes	No	Yes	No	No	Yes	Yes
2	14	0.750	88	13	8	0.04	M	Yes	No	Yes	No	No	Yes	Yes
80	14	0.797	69	15	4	0.17	F	Yes	Yes	No	No	No	No	No
98	14	0.880	158	33	24	0.31	F	Yes	No	No	No	No	No	Yes
113	14	0.714	28	20	0	0.09	F	No	No	No	No	No	No	No
181	14	0.857	42	20	38	0.08	M	No	No	No	No	No	No	No
246	14	0.804	51	29	44	0.2	M	No	No	No	No	No	No	No
248	14	0.833	36	28	14	0.15	F	No	No	No	No	No	No	No

286	14	0.667	9	6	0	0.09	F	No	No	No	No	No	No	No
366	14	0.878	41	0	34	0.27	F	No	Yes	No	No	No	Yes	No
387	14	0.873	63	40	38	0.58	M	No	Yes	No	No	No	No	No
536	14	0.714	14	12	0	0.24	F	Yes	No	No	No	No	Yes	No
227	15	0.857	77	24	22	0.35	F	No	No	No	No	No	No	No
308	16	0.611	18	15	6	0.21	F	No	No	No	No	No	Yes	No

Supplementary Table 4 | Putative post-baseline resistance mutations identified at Day 14 both at allele frequency above 5% and at consensus level (>50%). Detailed are mutations in NSP12/RdRp that are considered to be associated with drug resistance to molnupiravir based on comparative genomics using the SARS2-ResistanceDB (retrieved from https://github.com/ucl-pathgenomics/SARS2-ResistanceDB), and Spike mutations considered to be relevant for immune system escape retrieved from the Pokay database (https://github.com/nodrogluap/pokay).

GENE	Change Observed	Effect	AF	Number of participants
**NSP12**	V560L	Predicted Resistance Site	>5%	1
**NSP12**	V234I	Predicted Resistance Site	>50%	1
**S**	A831V	Immune Modulation/Escape	>5%	1
**S**	W64R	Immune Modulation/Escape	>5%	1
**S**	Q1010R	Immune Modulation/Escape	>5%	1
**S**	P1162S	Immune Modulation/Escape	>5%	2
**S**	V1128A	Immune Modulation/Escape	>5%	1
**S**	H146Y	Immune Modulation/Escape	>5%	1
**S**	C15Y	Immune Modulation/Escape	>5%	1
**S**	K150R	Immune Modulation/Escape	>50%	1
**S**	P681R	Immune Modulation/Escape	>50%	1
**S**	F490L	Immune Modulation/Escape	>50%	1
**S**	F490S	Immune Modulation/Escape	>50%	2

Supplementary Table 5 | Summary details of viral mutations for four post-molnupiravir treatment (>5 days) samples with culturable virus. Total numbers of post-baseline transitions and transversions at ≥5% allele frequency per sample and details of amino acid changes are shown.

Participant ID	Time (Days)	Number of transitions	Number of transversions	AA change from baseline	Positive Day 14 VL
**45**	Day 6	9	1	R408S, G295S, G133D, V1931I, A1812T, V699I, E381K, P34L, A143T, K160E	Yes
**85**	Day 14	79	2	R408S, N507I, A2129T, G251D, S1924N, S2535L, N192S, V2133I, A90V, T2306I, A2123T, H1613Y, S815P, D2544N, V84I, N43S, S201N, N2539D, T247I, V116A, A570T, R476H, A2575V, G120R, P1088L, D3511N, A1670V, N804D, V89I, D543N, C15Y, A217T, P820S, A607V, T49I, P3395H, A41V, D571N, G146D, V808I, T812I, P25S, V45I, L95S, V4I, M3280I, S778N, C1254Y, A2621T, C563Y, G11R, A1070T, V29I, M584V, T602I, P46S, P271L, R2818H, Y449H, A2745V, A656T, A4V, P792S, S461L, T334I, V1177I, S330L, R634H, E132K, K440R, T325I, H373Y, V1290I, A382V, P322S, H69Y, R3802H, G1068R, S173N, F13S, G2927S	Yes
**557**	Day 7	35	0	V544I, N3168D, S3195N, S34F, T2611I, E2617R, A3620V, K3630R, T3287I, V2816I, T2846I, T2906I, V244I, S50G, V70I, L7S, A64T, H245Y, E102K, V202I, V596M, V841I, M1057I, R173C, P461S, A1060T, D294N, T1322I, D909N, P1921L, F2182L, G2284S, T547I, V1765I, M1448I	Yes
**530**	Day 6	1	0	A181V	Yes

Supplementary Table 6 | New post-baseline, consensus level (AF >=50%), missense transition (G->A or C->T) mutations at Day 14 for molnupiravir-treated and Usual Care participants. Total molnupiravir: 224 and Usual Care: 6.

AA change	Mutation	Gene	Treatment
**D72N**	G->A	E	Molnupiravir
**L19F**	C->T	E	Molnupiravir
**A2T**	G->A	M	Molnupiravir
**R44K**	G->A	M	Molnupiravir
**D415N**	G->A	N	Molnupiravir
**G129D**	G->A	N	Molnupiravir
**H145Y**	C->T	N	Molnupiravir
**P365L**	C->T	N	Molnupiravir
**V246I**	G->A	N	Molnupiravir
**A130T**	G->A	NSP12	Molnupiravir
**A716V**	C->T	NSP12	Molnupiravir
**C22Y**	G->A	NSP12	Molnupiravir
**D303N**	G->A	NSP12	Molnupiravir
**G228D**	G->A	NSP12	Molnupiravir
**G228S**	G->A	NSP12	Molnupiravir
**H725Y**	C->T	NSP12	Molnupiravir
**H882Y**	C->T	NSP12	Molnupiravir
**P169S**	C->T	NSP12	Molnupiravir
**S15N**	G->A	NSP12	Molnupiravir
**S549N**	G->A	NSP12	Molnupiravir
**S778N**	G->A	NSP12	Molnupiravir
**T591I**	C->T	NSP12	Molnupiravir
**T76I**	C->T	NSP12	Molnupiravir
**V182I**	G->A	NSP12	Molnupiravir
**V234I**	G->A	NSP12	Molnupiravir
**V257I**	G->A	NSP12	Molnupiravir
**V359I**	G->A	NSP12	Molnupiravir
**V605I**	G->A	NSP12	Molnupiravir
**V905I**	G->A	NSP12	Molnupiravir
**A403T**	G->A	NSP13	Molnupiravir
**A454T**	G->A	NSP13	Molnupiravir
**A520T**	G->A	NSP13	Molnupiravir
**A568V**	C->T	NSP13	Molnupiravir
**D583N**	G->A	NSP13	Molnupiravir
**H290Y**	C->T	NSP13	Molnupiravir
**P491S**	C->T	NSP13	Molnupiravir
**P82L**	C->T	NSP13	Molnupiravir
**R339H**	G->A	NSP13	Molnupiravir
**R502H**	G->A	NSP13	Molnupiravir
**V340I**	G->A	NSP13	Molnupiravir

**V479I**	G->A	NSP13	Molnupiravir
**V521I**	G->A	NSP13	Molnupiravir
**V98I**	G->A	NSP13	Molnupiravir
**L56F**	C->T	NSP7	Molnupiravir
**A150V**	C->T	NSP8	Molnupiravir
**P178S**	C->T	NSP8	Molnupiravir
**T148I**	C->T	NSP8	Molnupiravir
**V115I**	G->A	NSP8	Molnupiravir
**V34I**	G->A	NSP8	Molnupiravir
**A8T**	G->A	ORF10	Molnupiravir
**V32I**	G->A	ORF10	Molnupiravir
**A1074T**	G->A	ORF1a	Molnupiravir
**A1397V**	C->T	ORF1a	Molnupiravir
**A1677T**	G->A	ORF1a	Molnupiravir
**A1679T**	G->A	ORF1a	Molnupiravir
**A1739V**	C->T	ORF1a	Molnupiravir
**A1809V**	C->T	ORF1a	Molnupiravir
**A1923T**	G->A	ORF1a	Molnupiravir
**A206T**	G->A	ORF1a	Molnupiravir
**A208T**	G->A	ORF1a	Molnupiravir
**A2142V**	C->T	ORF1a	Molnupiravir
**A2213T**	G->A	ORF1a	Molnupiravir
**A2279T**	G->A	ORF1a	Molnupiravir
**A2344T**	G->A	ORF1a	Molnupiravir
**A239V**	C->T	ORF1a	Molnupiravir
**A2584V**	C->T	ORF1a	Molnupiravir
**A2614T**	G->A	ORF1a	Molnupiravir
**A2614V**	C->T	ORF1a	Molnupiravir
**A2616T**	G->A	ORF1a	Molnupiravir
**A2745T**	G->A	ORF1a	Molnupiravir
**A2785T**	G->A	ORF1a	Molnupiravir
**A3023T**	G->A	ORF1a	Molnupiravir
**A3061T**	G->A	ORF1a	Molnupiravir
**A3220T**	G->A	ORF1a	Molnupiravir
**A3270T**	G->A	ORF1a	Molnupiravir
**A3571T**	G->A	ORF1a	Molnupiravir
**A3610V**	C->T	ORF1a	Molnupiravir
**A3615T**	G->A	ORF1a	Molnupiravir
**A3634T**	G->A	ORF1a	Molnupiravir
**A405T**	G->A	ORF1a	Molnupiravir
**A482T**	G->A	ORF1a	Molnupiravir
**A541T**	G->A	ORF1a	Molnupiravir
**A555T**	G->A	ORF1a	Molnupiravir
**A566T**	G->A	ORF1a	Molnupiravir

**A668V**	C->T	ORF1a	Molnupiravir
**A773V**	C->T	ORF1a	Molnupiravir
**A819T**	G->A	ORF1a	Molnupiravir
**A876V**	C->T	ORF1a	Molnupiravir
**A964T**	G->A	ORF1a	Molnupiravir
**A967T**	G->A	ORF1a	Molnupiravir
**C2180Y**	G->A	ORF1a	Molnupiravir
**C231Y**	G->A	ORF1a	Molnupiravir
**C3762Y**	G->A	ORF1a	Molnupiravir
**C506Y**	G->A	ORF1a	Molnupiravir
**C655Y**	G->A	ORF1a	Molnupiravir
**C800Y**	G->A	ORF1a	Molnupiravir
**D1179N**	G->A	ORF1a	Molnupiravir
**D1893N**	G->A	ORF1a	Molnupiravir
**D194N**	G->A	ORF1a	Molnupiravir
**D466N**	G->A	ORF1a	Molnupiravir
**D582N**	G->A	ORF1a	Molnupiravir
**E102K**	G->A	ORF1a	Molnupiravir
**E1086K**	G->A	ORF1a	Molnupiravir
**E2993K**	G->A	ORF1a	Molnupiravir
**E36K**	G->A	ORF1a	Molnupiravir
**E640K**	G->A	ORF1a	Molnupiravir
**E972K**	G->A	ORF1a	Molnupiravir
**G1408S**	G->A	ORF1a	Molnupiravir
**G150D**	G->A	ORF1a	Molnupiravir
**G2534S**	G->A	ORF1a	Molnupiravir
**G379E**	G->A	ORF1a	Molnupiravir
**G49S**	G->A	ORF1a	Molnupiravir
**G98D**	G->A	ORF1a	Molnupiravir
**H2799Y**	C->T	ORF1a	Molnupiravir
**L320F**	C->T	ORF1a	Molnupiravir
**L3829F**	C->T	ORF1a	Molnupiravir
**M2347I**	G->A	ORF1a	Molnupiravir
**M2719I**	G->A	ORF1a	Molnupiravir
**M3527I**	G->A	ORF1a	Molnupiravir
**M3621I**	G->A	ORF1a	Molnupiravir
**M3752I**	G->A	ORF1a	Molnupiravir
**M731I**	G->A	ORF1a	Molnupiravir
**M789I**	G->A	ORF1a	Molnupiravir
**M85I**	G->A	ORF1a	Molnupiravir
**P1786S**	C->T	ORF1a	Molnupiravir
**P2018S**	C->T	ORF1a	Molnupiravir
**P2110L**	C->T	ORF1a	Molnupiravir
**P2685S**	C->T	ORF1a	Molnupiravir

**P361S**	C->T	ORF1a	Molnupiravir
**P67S**	C->T	ORF1a	Molnupiravir
**P80S**	C->T	ORF1a	Molnupiravir
**P820L**	C->T	ORF1a	Molnupiravir
**P959S**	C->T	ORF1a	Molnupiravir
**R207C**	C->T	ORF1a	Molnupiravir
**R24H**	G->A	ORF1a	Molnupiravir
**R3662C**	C->T	ORF1a	Molnupiravir
**R3821K**	G->A	ORF1a	Molnupiravir
**R550H**	G->A	ORF1a	Molnupiravir
**R560H**	G->A	ORF1a	Molnupiravir
**S1924N**	G->A	ORF1a	Molnupiravir
**S1952L**	C->T	ORF1a	Molnupiravir
**S911F**	C->T	ORF1a	Molnupiravir
**T1004I**	C->T	ORF1a	Molnupiravir
**T1241I**	C->T	ORF1a	Molnupiravir
**T1429I**	C->T	ORF1a	Molnupiravir
**T1437I**	C->T	ORF1a	Molnupiravir
**T1678I**	C->T	ORF1a	Molnupiravir
**T1773I**	C->T	ORF1a	Molnupiravir
**T1890I**	C->T	ORF1a	Molnupiravir
**T2158I**	C->T	ORF1a	Molnupiravir
**T2264I**	C->T	ORF1a	Molnupiravir
**T265I**	C->T	ORF1a	Molnupiravir
**T2910I**	C->T	ORF1a	Molnupiravir
**T3032I**	C->T	ORF1a	Molnupiravir
**T3462I**	C->T	ORF1a	Molnupiravir
**T3579I**	C->T	ORF1a	Molnupiravir
**T484I**	C->T	ORF1a	Molnupiravir
**T568I**	C->T	ORF1a	Molnupiravir
**T602I**	C->T	ORF1a	Molnupiravir
**T821I**	C->T	ORF1a	Molnupiravir
**V1056M**	G->A	ORF1a	Molnupiravir
**V1063I**	G->A	ORF1a	Molnupiravir
**V1122I**	G->A	ORF1a	Molnupiravir
**V1393M**	G->A	ORF1a	Molnupiravir
**V2238I**	G->A	ORF1a	Molnupiravir
**V2496M**	G->A	ORF1a	Molnupiravir
**V2754I**	G->A	ORF1a	Molnupiravir
**V2955M**	G->A	ORF1a	Molnupiravir
**V3120I**	G->A	ORF1a	Molnupiravir
**V3166I**	G->A	ORF1a	Molnupiravir
**V3593I**	G->A	ORF1a	Molnupiravir
**V3751I**	G->A	ORF1a	Molnupiravir

**V3763I**	G->A	ORF1a	Molnupiravir
**V3847I**	G->A	ORF1a	Molnupiravir
**A100V**	C->T	ORF1ab	Molnupiravir
**A319V**	C->T	ORF1ab	Molnupiravir
**D827N**	G->A	ORF1ab	Molnupiravir
**E453K**	G->A	ORF1ab	Molnupiravir
**G416S**	G->A	ORF1ab	Molnupiravir
**P140S**	C->T	ORF1ab	Molnupiravir
**T472M**	C->T	ORF1ab	Molnupiravir
**V1012I**	G->A	ORF1ab	Molnupiravir
**V269I**	G->A	ORF1ab	Molnupiravir
**V421I**	G->A	ORF1ab	Molnupiravir
**V713I**	G->A	ORF1ab	Molnupiravir
**V841I**	G->A	ORF1ab	Molnupiravir
**A99T**	G->A	ORF3a	Molnupiravir
**D238N**	G->A	ORF3a	Molnupiravir
**E241K**	G->A	ORF3a	Molnupiravir
**G100D**	G->A	ORF3a	Molnupiravir
**G49D**	G->A	ORF3a	Molnupiravir
**H150Y**	C->T	ORF3a	Molnupiravir
**L147F**	C->T	ORF3a	Molnupiravir
**L65F**	C->T	ORF3a	Molnupiravir
**P104S**	C->T	ORF3a	Molnupiravir
**V168I**	G->A	ORF3a	Molnupiravir
**V259I**	G->A	ORF3a	Molnupiravir
**A105T**	G->A	ORF7a	Molnupiravir
**A79V**	C->T	ORF7a	Molnupiravir
**D51N**	G->A	ORF7a	Molnupiravir
**T61I**	C->T	ORF7a	Molnupiravir
**Q35***	C->T	ORF7b	Molnupiravir
**A15V**	C->T	ORF8	Molnupiravir
**A55T**	G->A	ORF8	Molnupiravir
**A1226V**	C->T	S	Molnupiravir
**A263T**	G->A	S	Molnupiravir
**A570V**	C->T	S	Molnupiravir
**A653V**	C->T	S	Molnupiravir
**A672T**	G->A	S	Molnupiravir
**A67T**	G->A	S	Molnupiravir
**A684T**	G->A	S	Molnupiravir
**A829V**	C->T	S	Molnupiravir
**C1236Y**	G->A	S	Molnupiravir
**D215N**	G->A	S	Molnupiravir
**G181E**	G->A	S	Molnupiravir
**H1088Y**	C->T	S	Molnupiravir

**Q954***	C->T	S	Molnupiravir
**R1185H**	G->A	S	Molnupiravir
**S1242N**	G->A	S	Molnupiravir
**S689N**	G->A	S	Molnupiravir
**T1160I**	C->T	S	Molnupiravir
**T547I**	C->T	S	Molnupiravir
**V1061I**	G->A	S	Molnupiravir
**V622I**	G->A	S	Molnupiravir
**R146H**	G->A	M	Usual Care
**P169S**	C->T	NSP12	Usual Care
**A336V**	C->T	NSP13	Usual Care
**T1754I**	C->T	ORF1a	Usual Care
**S1587L**	C->T	ORF1a	Usual Care
**V1291I**	G->A	ORF1a	Usual Care

Supplementary Table 7 | COG-ID for 1436 whole genome sequence uploaded to CLIMB database.

LOND-YYBE13K	LOND-YYBEON5	LOND-YYBEWZC	LOND-YYBEMI3	LOND-YYBEXJQ	LOND-YYBEKBD
LOND-YYBE18X	LOND-YYBEORZ	LOND-YYBEX15	LOND-YYBEMPK	LOND-YYBEXKC	LOND-YYBEKEI
LOND-YYBE1B5	LOND-YYBEOTH	LOND-YYBEX4M	LOND-YYBEMRH	LOND-YYBEXMK	LOND-YYBEKHC
LOND-YYBE1M8	LOND-YYBEP3X	LOND-YYBEX5J	LOND-YYBEO14	LOND-YYBEXPG	LOND-YYBEKI4
LOND-YYBE1N3	LOND-YYBEP5M	LOND-YYBEXD4	LOND-YYBEOGU	LOND-YYBEXRA	LOND-YYBEKSA
LOND-YYBE1RI	LOND-YYBEP6F	LOND-YYBEXNH	LOND-YYBEOWS	LOND-YYBEZ3F	LOND-YYBEMAU
LOND-YYBE1T4	LOND-YYBEP8W	LOND-YYBEXU3	LOND-YYBEP9D	LOND-YYBEZ5B	LOND-YYBEMGA
LOND-YYBE1Y7	LOND-YYBEPAT	LOND-YYBEXWZ	LOND-YYBEPDH	LOND-YYBEZ65	LOND-YYBEMQE
LOND-YYBEI8C	LOND-YYBEPT9	LOND-YYBE15G	LOND-YYBEPJO	LOND-YYBE16Y	LOND-YYBEOC8
LOND-YYBEIFO	LOND-YYBEPYN	LOND-YYBE1CF	LOND-YYBEPKQ	LOND-YYBE1HR	LOND-YYBEOIW
LOND-YYBEIPY	LOND-YYBEQ1H	LOND-YYBE1EP	LOND-YYBEPOB	LOND-YYBE1JM	LOND-YYBEOPF
LOND-YYBEIQ6	LOND-YYBEQ4C	LOND-YYBE1GT	LOND-YYBEPU5	LOND-YYBE1KJ	LOND-YYBEOS1
LOND-YYBEIWT	LOND-YYBEQ5K	LOND-YYBE31T	LOND-YYBEPW3	LOND-YYBE1PD	LOND-YYBEPHJ
LOND-YYBEIZM	LOND-YYBEQFZ	LOND-YYBE363	LOND-YYBEQ7G	LOND-YYBE1QB	LOND-YYBEPIZ
LOND-YYBEJ1B	LOND-YYBEQJX	LOND-YYBE3N1	LOND-YYBEQ8U	LOND-YYBE3C6	LOND-YYBEPMC
LOND-YYBEJ6J	LOND-YYBEQKP	LOND-YYBE3WP	LOND-YYBEQCJ	LOND-YYBE3GK	LOND-YYBEPSI
LOND-YYBEJ8A	LOND-YYBEQR3	LOND-YYBE3YS	LOND-YYBEQHE	LOND-YYBE3H7	LOND-YYBEQB9
LOND-YYBEJ98	LOND-YYBEQWA	LOND-YYBE4DX	LOND-YYBEQSW	LOND-YYBE3JR	LOND-YYBEQIS
LOND-YYBEJES	LOND-YYBESBZ	LOND-YYBE4FM	LOND-YYBESEJ	LOND-YYBE3OI	LOND-YYBEQN7
LOND-YYBEJOF	LOND-YYBETAP	LOND-YYBE4KB	LOND-YYBESIQ	LOND-YYBE3Q4	LOND-YYBEQP8
LOND-YYBEK4O	LOND-YYBETBH	LOND-YYBE4T1	LOND-YYBESMD	LOND-YYBE3RQ	LOND-YYBEQXD
LOND-YYBEK5Q	LOND-YYBETJC	LOND-YYBEAC9	LOND-YYBESOA	LOND-YYBE3SJ	LOND-YYBEQZ1
LOND-YYBEKG3	LOND-YYBETPR	LOND-YYBEAGM	LOND-YYBESP9	LOND-YYBE488	LOND-YYBES1W
LOND-YYBEKR7	LOND-YYBETQN	LOND-YYBEARX	LOND-YYBESWO	LOND-YYBE4BU	LOND-YYBES4R
LOND-YYBEKWH	LOND-YYBETY6	LOND-YYBEATW	LOND-YYBETDA	LOND-YYBE4C7	LOND-YYBES76
LOND-YYBEKYF	LOND-YYBEU1Z	LOND-YYBEAX3	LOND-YYBETHF	LOND-YYBE4XZ	LOND-YYBESAE
LOND-YYBEM9F	LOND-YYBEU5F	LOND-YYBEI95	LOND-YYBETKK	LOND-YYBEA3R	LOND-YYBESKF
LOND-YYBEMBN	LOND-YYBEU69	LOND-YYBEIAJ	LOND-YYBETO7	LOND-YYBEABI	LOND-YYBESRT
LOND-YYBEMHM	LOND-YYBEU7B	LOND-YYBEIEK	LOND-YYBEU97	LOND-YYBEAH6	LOND-YYBET6B
LOND-YYBEMJ1	LOND-YYBEUB4	LOND-YYBEIHB	LOND-YYBEUDS	LOND-YYBEAKD	LOND-YYBET7D
LOND-YYBEMKO	LOND-YYBEUJK	LOND-YYBEIJE	LOND-YYBEUMG	LOND-YYBEASK	LOND-YYBETFW

LOND-YYBEMSZ	LOND-YYBEUKE	LOND-YYBEISP	LOND-YYBEUQY	LOND-YYBEAWQ	LOND-YYBETRS
LOND-YYBEMU7	LOND-YYBEUNA	LOND-YYBEJ3U	LOND-YYBEURW	LOND-YYBEI1I	LOND-YYBETUZ
LOND-YYBEMZI	LOND-YYBEUYH	LOND-YYBEJ4T	LOND-YYBEUSX	LOND-YYBEIBA	LOND-YYBEUCR
LOND-YYBEO3C	LOND-YYBEW66	LOND-YYBEJMO	LOND-YYBEW9H	LOND-YYBEIKG	LOND-YYBEUF1
LOND-YYBEOAQ	LOND-YYBEW7Y	LOND-YYBEJU9	LOND-YYBEWAK	LOND-YYBEIO3	LOND-YYBEW3E
LOND-YYBEOJP	LOND-YYBEWGX	LOND-YYBEKX8	LOND-YYBEWTA	LOND-YYBEIY4	LOND-YYBEW4G
LOND-YYBEOKM	LOND-YYBEWQ9	LOND-YYBEM19	LOND-YYBEX6D	LOND-YYBEK7K	LOND-YYBEWB3
LOND-YYBEOMJ	LOND-YYBEWX7	LOND-YYBEMF4	LOND-YYBEXH8	LOND-YYBEK8Z	LOND-YYBEWJJ
LOND-YYBEWNZ	LOND-YYBJE8Z	LOND-YYBJPFS	LOND-YYBJGAS	LOND-YYBJRHO	LOND-YYBJCQF
LOND-YYBEX9B	LOND-YYBJE9G	LOND-YYBJPII	LOND-YYBJGEZ	LOND-YYBJRPX	LOND-YYBJDBE
LOND-YYBEXFS	LOND-YYBJEAW	LOND-YYBJPQR	LOND-YYBJGS4	LOND-YYBJRSH	LOND-YYBJDC1
LOND-YYBEXII	LOND-YYBJEG3	LOND-YYBJPWZ	LOND-YYBJK3O	LOND-YYBJRTG	LOND-YYBJENB
LOND-YYBEXSU	LOND-YYBJEOR	LOND-YYBJQC8	LOND-YYBJK8I	LOND-YYBE3Z8	LOND-YYBJESA
LOND-YYBEXZT	LOND-YYBJEU6	LOND-YYBJQO6	LOND-YYBJKN9	LOND-YYBE456	LOND-YYBJF5T
LOND-YYBEZ8K	LOND-YYBJEX8	LOND-YYBJQXB	LOND-YYBJKTY	LOND-YYBE4P5	LOND-YYBJGKU
LOND-YYBEZBS	LOND-YYBJF1D	LOND-YYBJRDD	LOND-YYBJKUH	LOND-YYBE755	LOND-YYBJK9R
LOND-YYBEZD1	LOND-YYBJF3I	LOND-YYBJRJZ	LOND-YYBJKYD	LOND-YYBE9YO	LOND-YYBJKJT
LOND-YYBEZJG	LOND-YYBJFCO	LOND-YYBJRMU	LOND-YYBJKZW	LOND-YYBEA7H	LOND-YYBJME1
LOND-YYBEZOZ	LOND-YYBJFDN	LOND-YYBJRNF	LOND-YYBJM4P	LOND-YYBEH5H	LOND-YYBJMPE
LOND-YYBEZQH	LOND-YYBJFKW	LOND-YYBJRQP	LOND-YYBJM78	LOND-YYBESNI	LOND-YYBJNJ3
LOND-YYBEZSM	LOND-YYBJFO8	LOND-YYBJRWY	LOND-YYBJMFA	LOND-YYBEUT3	LOND-YYBJNTE
LOND-YYBEZWX	LOND-YYBJFQC	LOND-YYBJRXM	LOND-YYBJMGS	LOND-YYBEZ1U	LOND-YYBJNWN
LOND-YYBE1AC	LOND-YYBJFW9	LOND-YYBESFX	LOND-YYBJMHJ	LOND-YYBJ8W7	LOND-YYBJPMK
LOND-YYBEO9Y	LOND-YYBJFYE	LOND-YYBJ84T	LOND-YYBJMIZ	LOND-YYBJ8ZZ	LOND-YYBJPXN
LOND-YYBESHY	LOND-YYBJGND	LOND-YYBJ87M	LOND-YYBJMW3	LOND-YYBJBRR	LOND-YYBJQMJ
LOND-YYBEX3P	LOND-YYBJGUY	LOND-YYBJ8NN	LOND-YYBJN5W	LOND-YYBJC5K	LOND-YYBE5AD
LOND-YYBJ81B	LOND-YYBJGXJ	LOND-YYBJ8R6	LOND-YYBJN7O	LOND-YYBJCZ1	LOND-YYBJD5U
LOND-YYBJ8CQ	LOND-YYBJGY8	LOND-YYBJB7T	LOND-YYBJNRD	LOND-YYBJGHQ	LOND-YYBJD6P
LOND-YYBJ8HP	LOND-YYBJK4Q	LOND-YYBJC8U	LOND-YYBJNXP	LOND-YYBJGOG	LOND-YYBJEQJ
LOND-YYBJ8JW	LOND-YYBJK5C	LOND-YYBJCMM	LOND-YYBJP4M	LOND-YYBJGPP	LOND-YYBJFNR
LOND-YYBJ8OF	LOND-YYBJK7E	LOND-YYBJCOY	LOND-YYBJPGW	LOND-YYBJM9D	LOND-YYBJG1N
LOND-YYBJ8QK	LOND-YYBJKCM	LOND-YYBJCR3	LOND-YYBJPJQ	LOND-YYBJMKQ	LOND-YYBJGIH
LOND-YYBJ8TD	LOND-YYBJKD7	LOND-YYBJCWA	LOND-YYBJPKC	LOND-YYBJMR4	LOND-YYBJKGZ

LOND-YYBJB9P	LOND-YYBJKIA	LOND-YYBJD9M	LOND-YYBJPRA	LOND-YYBJNBJ	LOND-YYBJKQ8
LOND-YYBJC3Q	LOND-YYBJM6F	LOND-YYBJDHT	LOND-YYBJPSU	LOND-YYBJP5J	LOND-YYBJNQX
LOND-YYBJC9N	LOND-YYBJM8W	LOND-YYBJDQQ	LOND-YYBJPT7	LOND-YYBJPAX	LOND-YYBJO8X
LOND-YYBJCB9	LOND-YYBJMBY	LOND-YYBJDRN	LOND-YYBJPU3	LOND-YYBJQ8T	LOND-YYBJOAC
LOND-YYBJCIS	LOND-YYBJMCK	LOND-YYBJDUD	LOND-YYBJPZT	LOND-YYBJQD3	LOND-YYBJOJM
LOND-YYBJCSW	LOND-YYBJMOB	LOND-YYBJE4O	LOND-YYBJQB7	LOND-YYBE9OX	LOND-YYBJON3
LOND-YYBJCT6	LOND-YYBJMSI	LOND-YYBJEHC	LOND-YYBJQFI	LOND-YYBEAP7	LOND-YYBJOQB
LOND-YYBJD8H	LOND-YYBJMT9	LOND-YYBJEMX	LOND-YYBJQJP	LOND-YYBEI79	LOND-YYBJOY7
LOND-YYBJDA3	LOND-YYBJMXR	LOND-YYBJER7	LOND-YYBJQPF	LOND-YYBESQ7	LOND-YYBJQHG
LOND-YYBJDE4	LOND-YYBJN9C	LOND-YYBJETN	LOND-YYBJQRZ	LOND-YYBETZX	LOND-YYBJQWS
LOND-YYBJDI9	LOND-YYBJNA4	LOND-YYBJEYF	LOND-YYBJQY9	LOND-YYBEUHD	LOND-YYBJRE3
LOND-YYBJDKS	LOND-YYBJP15	LOND-YYBJF6M	LOND-YYBJR4W	LOND-YYBEZRO	LOND-YYBJX13
LOND-YYBJE1Y	LOND-YYBJP3P	LOND-YYBJF84	LOND-YYBJR6C	LOND-YYBJ8K1	LOND-YYBJX6B
LOND-YYBJE6E	LOND-YYBJP9B	LOND-YYBJG9E	LOND-YYBJRAA	LOND-YYBJC7G	LOND-YYBJXAP
LOND-YYBJXBH	LOND-YYBJB5I	LOND-YYBJNE5	LOND-YYBJA3N	LOND-YYBJQAQ	LOND-YYBJXQN
LOND-YYBJXFW	LOND-YYBJBCW	LOND-YYBJNH1	LOND-YYBJA4Y	LOND-YYBJQS1	LOND-YYBJXRS
LOND-YYBJXKK	LOND-YYBJBEH	LOND-YYBJNIY	LOND-YYBJAQ3	LOND-YYBJQUA	LOND-YYBJXT5
LOND-YYBJXN4	LOND-YYBJBFN	LOND-YYBJNKZ	LOND-YYBJH1C	LOND-YYBJS11	LOND-YYBJXUZ
LOND-YYBJXO7	LOND-YYBJBHU	LOND-YYBJNYM	LOND-YYBJH36	LOND-YYBJS3R	LOND-YYBJXZX
LOND-YYBJXPR	LOND-YYBJBKA	LOND-YYBJR3S	LOND-YYBJH6W	LOND-YYBJS4N	LOND-YYBJZ63
LOND-YYBJXST	LOND-YYBJD3Z	LOND-YYBJR7Q	LOND-YYBJHF8	LOND-YYBJS7H	LOND-YYBJZC6
LOND-YYBJXWI	LOND-YYBJD7X	LOND-YYBJRCT	LOND-YYBJHGF	LOND-YYBJSBI	LOND-YYBJZGK
LOND-YYBJYGB	LOND-YYBJDFY	LOND-YYBJRI6	LOND-YYBJHM5	LOND-YYBJSC9	LOND-YYBJZJR
LOND-YYBE4HH	LOND-YYBJDJA	LOND-YYBJRKI	LOND-YYBJHTQ	LOND-YYBJSE8	LOND-YYBJZKN
LOND-YYBE573	LOND-YYBJDMW	LOND-YYBJROE	LOND-YYBJHYT	LOND-YYBJSKD	LOND-YYBJZMY
LOND-YYBE5GE	LOND-YYBJDPO	LOND-YYBJRYJ	LOND-YYBJHZN	LOND-YYBJSOS	LOND-YYBJZQ4
LOND-YYBE64H	LOND-YYBJDS7	LOND-YYBJYMZ	LOND-YYBJTDS	LOND-YYBJSQ5	LOND-YYBJZRQ
LOND-YYBE6BX	LOND-YYBJDT8	LOND-YYBJYPU	LOND-YYBJU67	LOND-YYBJT3J	LOND-YYBJZTU
LOND-YYBE6K7	LOND-YYBJDWB	LOND-YYBJYSY	LOND-YYBJUY4	LOND-YYBJT5F	LOND-YYBJZUX
LOND-YYBE7MH	LOND-YYBJDXC	LOND-YYBJYTK	LOND-YYBJX3M	LOND-YYBJT97	LOND-YYBJZWP
LOND-YYBE7NQ	LOND-YYBJDYK	LOND-YYBJYWR	LOND-YYBJ1GX	LOND-YYBJTAM	LOND-YYBJ31X
LOND-YYBE7PA	LOND-YYBJEBD	LOND-YYBJ3QA	LOND-YYBJ1Q9	LOND-YYBJTGO	LOND-YYBJ3AD
LOND-YYBE9DK	LOND-YYBJEPM	LOND-YYBJ3RC	LOND-YYBJ1SQ	LOND-YYBJTJK	LOND-YYBJ3B1

LOND-YYBE9EY	LOND-YYBJEZS	LOND-YYBJ43Y	LOND-YYBJ3ER	LOND-YYBJTO5	LOND-YYBJ3CH
LOND-YYBE9KH	LOND-YYBJFGH	LOND-YYBJ4IE	LOND-YYBJ3UP	LOND-YYBJTWU	LOND-YYBJ3DQ
LOND-YYBE9ZB	LOND-YYBJFHX	LOND-YYBJ4SG	LOND-YYBJABU	LOND-YYBJUBA	LOND-YYBJ3KY
LOND-YYBEH3Y	LOND-YYBJFJS	LOND-YYBJ4TO	LOND-YYBJHUK	LOND-YYBJUEK	LOND-YYBJ3M6
LOND-YYBEHDP	LOND-YYBJFM1	LOND-YYBJ4ZR	LOND-YYBJI5B	LOND-YYBJUFO	LOND-YYBJ3NO
LOND-YYBEHFJ	LOND-YYBJGR9	LOND-YYBJ51P	LOND-YYBJIEE	LOND-YYBJUHB	LOND-YYBJ3ZF
LOND-YYBEHK9	LOND-YYBJGW6	LOND-YYBJ5DC	LOND-YYBJIRO	LOND-YYBJUPY	LOND-YYBJ41Q
LOND-YYBEHUC	LOND-YYBJK16	LOND-YYBJ61K	LOND-YYBJIYA	LOND-YYBJUWT	LOND-YYBJ45H
LOND-YYBEJPC	LOND-YYBJKA1	LOND-YYBJ63H	LOND-YYBJO1A	LOND-YYBJW5N	LOND-YYBJ46S
LOND-YYBEJR6	LOND-YYBJKBB	LOND-YYBJ6IR	LOND-YYBJO3K	LOND-YYBJW8M	LOND-YYBJ47A
LOND-YYBEPXR	LOND-YYBJKHK	LOND-YYBJ6PI	LOND-YYBJOEP	LOND-YYBJWFX	LOND-YYBJ48F
LOND-YYBJ8AI	LOND-YYBJKMP	LOND-YYBJ76U	LOND-YYBJOGT	LOND-YYBJWMD	LOND-YYBJ4BT
LOND-YYBJ8DY	LOND-YYBJKPJ	LOND-YYBJ78N	LOND-YYBJOHR	LOND-YYBJWP9	LOND-YYBJ4ED
LOND-YYBJ8FH	LOND-YYBJKXF	LOND-YYBJ7DK	LOND-YYBJOM8	LOND-YYBJWQ7	LOND-YYBJ4G8
LOND-YYBJ8G4	LOND-YYBJMYN	LOND-YYBJ7IF	LOND-YYBJOPD	LOND-YYBJWSC	LOND-YYBJ4H4
LOND-YYBJ8MO	LOND-YYBJN3A	LOND-YYBJ7J6	LOND-YYBJOX9	LOND-YYBJX4J	LOND-YYBJ4P3
LOND-YYBJ8PC	LOND-YYBJN4S	LOND-YYBJ7KH	LOND-YYBJOZQ	LOND-YYBJX99	LOND-YYBJ4QZ
LOND-YYBJ8XE	LOND-YYBJN6Q	LOND-YYBJ7OX	LOND-YYBJQ4K	LOND-YYBJXEQ	LOND-YYBJ4WK
LOND-YYBJ8YG	LOND-YYBJN87	LOND-YYBJ7RE	LOND-YYBJQ7R	LOND-YYBJXIU	LOND-YYBJ4XI
LOND-YYBJB18	LOND-YYBJNCU	LOND-YYBJ7SD	LOND-YYBJQ9Y	LOND-YYBJXME	LOND-YYBJ4YU
LOND-YYBJ547	LOND-YYBJAZG	LOND-YYBJ5TX	LOND-YYBJNG9	LOND-YYBJS64	LOND-YYBJWZK
LOND-YYBJ5AB	LOND-YYBJH4H	LOND-YYBJ5UM	LOND-YYBJNS6	LOND-YYBJSGM	LOND-YYBJXG1
LOND-YYBJ5EN	LOND-YYBJH54	LOND-YYBJ5WJ	LOND-YYBJP6D	LOND-YYBJSIC	LOND-YYBJZ59
LOND-YYBJ5GG	LOND-YYBJH7S	LOND-YYBJ6A6	LOND-YYBJYUG	LOND-YYBJSJF	LOND-YYBJZAF
LOND-YYBJ5H3	LOND-YYBJH91	LOND-YYBJ6CZ	LOND-YYBJ13E	LOND-YYBJSMB	LOND-YYBJZBW
LOND-YYBJ5OT	LOND-YYBJHBX	LOND-YYBJ6J7	LOND-YYBJ15R	LOND-YYBJSP7	LOND-YYBJZIM
LOND-YYBJ5RK	LOND-YYBJHDM	LOND-YYBJ6K5	LOND-YYBJ1DI	LOND-YYBJSRX	LOND-YYBJ18P
LOND-YYBJ5XW	LOND-YYBJHEB	LOND-YYBJ6M3	LOND-YYBJ1IO	LOND-YYBJSTW	LOND-YYBJ19H
LOND-YYBJ6DJ	LOND-YYBJHIG	LOND-YYBJ737	LOND-YYBJ1JJ	LOND-YYBJSWQ	LOND-YYBJ1B3
LOND-YYBJ6E9	LOND-YYBJHJ9	LOND-YYBJ7A9	LOND-YYBJ1RU	LOND-YYBJSX3	LOND-YYBJ1CD
LOND-YYBJ6FF	LOND-YYBJHQI	LOND-YYBJ7GR	LOND-YYBJ1UW	LOND-YYBJSZE	LOND-YYBJ1FT
LOND-YYBJ6OQ	LOND-YYBJHRJ	LOND-YYBJ7M4	LOND-YYBJ1W1	LOND-YYBJT69	LOND-YYBJ1O4
LOND-YYBJ6QU	LOND-YYBJHWE	LOND-YYBJ7W8	LOND-YYBJ1ZC	LOND-YYBJTF1	LOND-YYBJ1X7

LOND-YYBJ6R8	LOND-YYBJHXU	LOND-YYBJ7YO	LOND-YYBJAC7	LOND-YYBJTHD	LOND-YYBJC1H
LOND-YYBJ6SN	LOND-YYBJID1	LOND-YYBJA56	LOND-YYBJADX	LOND-YYBJTNA	LOND-YYBJC4C
LOND-YYBJ6TC	LOND-YYBJTRW	LOND-YYBJA9S	LOND-YYBJAJD	LOND-YYBJTSX	LOND-YYBJCD5
LOND-YYBJ6UE	LOND-YYBJZ1T	LOND-YYBJAUQ	LOND-YYBJARP	LOND-YYBJTX6	LOND-YYBJEKT
LOND-YYBJ6WG	LOND-YYBJZFC	LOND-YYBJH8D	LOND-YYBJI1U	LOND-YYBJTZP	LOND-YYBJM3X
LOND-YYBJ6XT	LOND-YYBJZPH	LOND-YYBJHAY	LOND-YYBJI4D	LOND-YYBJU1I	LOND-YYBJOCF
LOND-YYBJ745	LOND-YYBJ1NZ	LOND-YYBJHK7	LOND-YYBJI77	LOND-YYBJU79	LOND-YYBJOFU
LOND-YYBJ79T	LOND-YYBJ3FK	LOND-YYBJHOO	LOND-YYBJIA8	LOND-YYBJU8C	LOND-YYBJOSO
LOND-YYBJ7BQ	LOND-YYBJ3GE	LOND-YYBJHSR	LOND-YYBJIBS	LOND-YYBJUDW	LOND-YYBJOT4
LOND-YYBJ7CA	LOND-YYBJ3H5	LOND-YYBJQ14	LOND-YYBJIFQ	LOND-YYBJUIX	LOND-YYBJOWW
LOND-YYBJ7EY	LOND-YYBJ3P4	LOND-YYBJT48	LOND-YYBJIH9	LOND-YYBJUKG	LOND-YYBJPEO
LOND-YYBJ7HZ	LOND-YYBJ4AN	LOND-YYBJXDA	LOND-YYBJIJG	LOND-YYBJUQ6	LOND-YYBJQ3C
LOND-YYBJ7NC	LOND-YYBJ4DP	LOND-YYBJZ8E	LOND-YYBJIKR	LOND-YYBJUR1	LOND-YYBJQ5E
LOND-YYBJ7PS	LOND-YYBJ4JB	LOND-YYBE3KN	LOND-YYBJINW	LOND-YYBJUSP	LOND-YYBJQ6N
LOND-YYBJ7QW	LOND-YYBJ4K9	LOND-YYBE5CH	LOND-YYBJIP6	LOND-YYBJW4R	LOND-YYBJQIW
LOND-YYBJ7TP	LOND-YYBJ4M7	LOND-YYBE5JN	LOND-YYBJIUT	LOND-YYBJW6H	LOND-YYBJQN5
LOND-YYBJ7UJ	LOND-YYBJ4NX	LOND-YYBE5WM	LOND-YYBJIWX	LOND-YYBJWAE	LOND-YYBJQTH
LOND-YYBJ7X1	LOND-YYBJ4O1	LOND-YYBE7TX	LOND-YYBJIX4	LOND-YYBJWBZ	LOND-YYBJT1Z
LOND-YYBJA1O	LOND-YYBJ56I	LOND-YYBE96U	LOND-YYBJIZJ	LOND-YYBJWCB	LOND-YYBJT8Q
LOND-YYBJA6A	LOND-YYBJ59U	LOND-YYBEHP3	LOND-YYBJO4E	LOND-YYBJWHY	LOND-YYBJTB4
LOND-YYBJA74	LOND-YYBJ5BO	LOND-YYBJ8ES	LOND-YYBJO5G	LOND-YYBJWIQ	LOND-YYBJTCR
LOND-YYBJAIK	LOND-YYBJ5C4	LOND-YYBJBXQ	LOND-YYBJO6Y	LOND-YYBJWKF	LOND-YYBJTKE
LOND-YYBJANT	LOND-YYBJ5JY	LOND-YYBJGDB	LOND-YYBJO7N	LOND-YYBJWTS	LOND-YYBJTMG
LOND-YYBJASE	LOND-YYBJ5K6	LOND-YYBJKON	LOND-YYBJOKJ	LOND-YYBJWU1	LOND-YYBJTUI
LOND-YYBJAT1	LOND-YYBJ5MH	LOND-YYBJKR5	LOND-YYBJPYY	LOND-YYBJWX5	LOND-YYBJTYH
LOND-YYBJAWC	LOND-YYBJ5NQ	LOND-YYBJMU5	LOND-YYBJQZO	LOND-YYBJWY3	LOND-YYBJU5D
LOND-YYBJUCN	LOND-YYBJAXZ	LOND-YYBE1OH	LOND-YYBEMCC	LOND-YYBEWDI	LOND-YYBE7OT
LOND-YYBJUJE	LOND-YYBJAYI	LOND-YYBE1X9	LOND-YYBEMD6	LOND-YYBEWHN	LOND-YYBE7QS
LOND-YYBJUNS	LOND-YYBJHC3	LOND-YYBE1ZQ	LOND-YYBEMEW	LOND-YYBEWIO	LOND-YYBE7WJ
LOND-YYBJUTZ	LOND-YYBJHPZ	LOND-YYBEICN	LOND-YYBEMTB	LOND-YYBEWK8	LOND-YYBE91M
LOND-YYBJWDU	LOND-YYBJI3F	LOND-YYBEIDW	LOND-YYBEMW5	LOND-YYBEWSQ	LOND-YYBE97I
LOND-YYBJWEJ	LOND-YYBJI65	LOND-YYBEIR1	LOND-YYBEMYR	LOND-YYBEWUW	LOND-YYBE98N
LOND-YYBJWGP	LOND-YYBJI8K	LOND-YYBEIUU	LOND-YYBEO6N	LOND-YYBEX81	LOND-YYBE9FG

LOND-YYBJWRT	LOND-YYBJICY	LOND-YYBEJ5X	LOND-YYBEO8T	LOND-YYBEXAX	LOND-YYBE9PS
LOND-YYBJX7D	LOND-YYBJIOZ	LOND-YYBEJ7M	LOND-YYBEOB7	LOND-YYBEXB6	LOND-YYBE9RE
LOND-YYBJX8O	LOND-YYBJIQH	LOND-YYBEJAI	LOND-YYBEOFI	LOND-YYBEXT7	LOND-YYBE9W8
LOND-YYBJXHF	LOND-YYBJO96	LOND-YYBEJCQ	LOND-YYBEOQD	LOND-YYBE196	LOND-YYBEA11
LOND-YYBJXJC	LOND-YYBJOB5	LOND-YYBEJDY	LOND-YYBEOXB	LOND-YYBE1I1	LOND-YYBEA4N
LOND-YYBJ14G	LOND-YYBJOI1	LOND-YYBEJFH	LOND-YYBEOZO	LOND-YYBE1US	LOND-YYBEH46
LOND-YYBJ166	LOND-YYBJORI	LOND-YYBEJG4	LOND-YYBEP4P	LOND-YYBE1WW	LOND-YYBEHC5
LOND-YYBJ1EM	LOND-YYBJSAG	LOND-YYBEJHP	LOND-YYBEP78	LOND-YYBE375	LOND-YYBEHJB
LOND-YYBJ1HN	LOND-YYBJSDT	LOND-YYBEJI5	LOND-YYBEPCK	LOND-YYBE3AF	LOND-YYBEHM7
LOND-YYBJ1K8	LOND-YYBJSFP	LOND-YYBEJQK	LOND-YYBEPE1	LOND-YYBE3BW	LOND-YYBEHO1
LOND-YYBJ1MF	LOND-YYBJSH6	LOND-YYBEJS3	LOND-YYBEPFA	LOND-YYBE3IM	LOND-YYBEHRM
LOND-YYBJ1TA	LOND-YYBJSNU	LOND-YYBEJTD	LOND-YYBEPGS	LOND-YYBE41O	LOND-YYBEHSG
LOND-YYBJ36Z	LOND-YYBJT7B	LOND-YYBEJW7	LOND-YYBEPZU	LOND-YYBE43N	LOND-YYBEHXI
LOND-YYBJ373	LOND-YYBJU95	LOND-YYBEJXE	LOND-YYBEQ3Q	LOND-YYBE474	LOND-YYBEI4F
LOND-YYBJ3IJ	LOND-YYBJUAJ	LOND-YYBEJZZ	LOND-YYBEQ9N	LOND-YYBE4JD	LOND-YYBEI67
LOND-YYBJ3JN	LOND-YYBJUMR	LOND-YYBEK1Y	LOND-YYBEQAO	LOND-YYBE4Q3	LOND-YYBEIMR
LOND-YYBJ3TT	LOND-YYBJUXH	LOND-YYBEK31	LOND-YYBEQET	LOND-YYBE4ZG	LOND-YYBEK6E
LOND-YYBJ3XS	LOND-YYBJUZM	LOND-YYBEK9G	LOND-YYBEQMM	LOND-YYBE5B1	LOND-YYBEM3T
LOND-YYBJ3YW	LOND-YYBJW1W	LOND-YYBEKCP	LOND-YYBEQOY	LOND-YYBE5FK	LOND-YYBEM4X
LOND-YYBJ4C5	LOND-YYBJW3G	LOND-YYBEKD9	LOND-YYBEQT6	LOND-YYBE5H5	LOND-YYBEO7R
LOND-YYBJ4RM	LOND-YYBJW76	LOND-YYBEKF5	LOND-YYBET3M	LOND-YYBE636	LOND-YYBEQD5
LOND-YYBJ5I8	LOND-YYBJWJ8	LOND-YYBEKJU	LOND-YYBET8O	LOND-YYBE654	LOND-YYBES3G
LOND-YYBJ5PA	LOND-YYBJWOA	LOND-YYBEKMX	LOND-YYBET99	LOND-YYBE691	LOND-YYBES6H
LOND-YYBJ5QS	LOND-YYBJZ3D	LOND-YYBEKNB	LOND-YYBETEQ	LOND-YYBE6F8	LOND-YYBESGP
LOND-YYBJ5ZD	LOND-YYBJZ4B	LOND-YYBEKOR	LOND-YYBETME	LOND-YYBE6HA	LOND-YYBESJ8
LOND-YYBJA88	LOND-YYBJZEG	LOND-YYBEKPM	LOND-YYBETST	LOND-YYBE6M5	LOND-YYBEUAM
LOND-YYBJAFM	LOND-YYBJZH7	LOND-YYBEKQJ	LOND-YYBEU8Q	LOND-YYBE6PZ	LOND-YYBEUGO
LOND-YYBJAGJ	LOND-YYBJZN1	LOND-YYBEKTN	LOND-YYBEUWU	LOND-YYBE6TQ	LOND-YYBEUZP
LOND-YYBJAHH	LOND-YYBJZOI	LOND-YYBEKU6	LOND-YYBEUX6	LOND-YYBE7C4	LOND-YYBEW1S
LOND-YYBJAKB	LOND-YYBJZSJ	LOND-YYBEKZS	LOND-YYBEW5R	LOND-YYBE7DC	LOND-YYBEWFT
LOND-YYBJAM9	LOND-YYBJZXA	LOND-YYBEM5P	LOND-YYBEW8P	LOND-YYBE7EN	LOND-YYBEXO9
LOND-YYBJAOW	LOND-YYBE1FU	LOND-YYBEM7J	LOND-YYBEWCD	LOND-YYBE7GG	LOND-YYBEXQR
LOND-YYBEZCY	LOND-YYBE7SF	LOND-YYBJYIN	LOND-YYBESCB	LOND-YYBE3PH	LOND-YYBJE31

LOND-YYBEZUT	LOND-YYBE9A9	LOND-YYBE3MY	LOND-YYBESTS	LOND-YYBE3UX	LOND-YYBJE5Q
LOND-YYBJ3WM	LOND-YYBE9M4	LOND-YYBE4AR	LOND-YYBESU1	LOND-YYBE3XA	LOND-YYBJED9
LOND-YYBJ4FJ	LOND-YYBE9QW	LOND-YYBE4M9	LOND-YYBESX5	LOND-YYBE4NT	LOND-YYBJEI4
LOND-YYBJBUF	LOND-YYBE9SD	LOND-YYBE4WC	LOND-YYBETWI	LOND-YYBE4SE	LOND-YYBJEJU
LOND-YYBJYE7	LOND-YYBE9TP	LOND-YYBE51X	LOND-YYBEU3J	LOND-YYBE5ER	LOND-YYBJF9J
LOND-YYBJYFD	LOND-YYBEA9A	LOND-YYBE56Z	LOND-YYBEZA8	LOND-YYBE5KY	LOND-YYBJFI7
LOND-YYBJYOC	LOND-YYBEADT	LOND-YYBE5IJ	LOND-YYBEZEE	LOND-YYBE5TT	LOND-YYBJFTF
LOND-YYBJYQT	LOND-YYBEAIC	LOND-YYBE5NO	LOND-YYBEZH9	LOND-YYBE61C	LOND-YYBJFUB
LOND-YYBE1DZ	LOND-YYBEAMB	LOND-YYBE5QA	LOND-YYBEZNW	LOND-YYBE6IG	LOND-YYBJFZ3
LOND-YYBE1SO	LOND-YYBEAOS	LOND-YYBE5RC	LOND-YYBEZX4	LOND-YYBE6WE	LOND-YYBJG5O
LOND-YYBE3EG	LOND-YYBEH1Q	LOND-YYBE5UP	LOND-YYBJB33	LOND-YYBE7AB	LOND-YYBJGJI
LOND-YYBE3FC	LOND-YYBEH6S	LOND-YYBE6AY	LOND-YYBJB4Z	LOND-YYBE9BQ	LOND-YYBJGMT
LOND-YYBE3TU	LOND-YYBEH7A	LOND-YYBE6EB	LOND-YYBJBDG	LOND-YYBE9HZ	LOND-YYBJGTR
LOND-YYBE46A	LOND-YYBEH9W	LOND-YYBE6RJ	LOND-YYBJBIB	LOND-YYBE9IF	LOND-YYBJGZA
LOND-YYBE4GJ	LOND-YYBEHAN	LOND-YYBE6SR	LOND-YYBJBJ4	LOND-YYBE9J6	LOND-YYBJMDH
LOND-YYBE4IK	LOND-YYBEHIE	LOND-YYBE6UK	LOND-YYBJ1AK	LOND-YYBEA64	LOND-YYBJMJO
LOND-YYBE4OW	LOND-YYBEHNX	LOND-YYBE6YT	LOND-YYBJBS9	LOND-YYBEANU	LOND-YYBJMZU
LOND-YYBE4RP	LOND-YYBEHWK	LOND-YYBE79U	LOND-YYBJBWD	LOND-YYBEAQ5	LOND-YYBJNUR
LOND-YYBE59I	LOND-YYBEHZR	LOND-YYBE7I8	LOND-YYBJBYC	LOND-YYBEAZE	LOND-YYBJP81
LOND-YYBE5DQ	LOND-YYBEI5D	LOND-YYBE7UM	LOND-YYBJD4I	LOND-YYBEHBT	LOND-YYBJPH8
LOND-YYBE5M6	LOND-YYBEINS	LOND-YYBE7XW	LOND-YYBJFBG	LOND-YYBEQ6R	LOND-YYBJPO9
LOND-YYBE5P4	LOND-YYBEMXG	LOND-YYBE7Y1	LOND-YYBJFPQ	LOND-YYBESY3	LOND-YYBJQKM
LOND-YYBE5XS	LOND-YYBEO5E	LOND-YYBE9CA	LOND-YYBJFXK	LOND-YYBET4J	LOND-YYBJS9A
LOND-YYBE5ZF	LOND-YYBEOHG	LOND-YYBE9GR	LOND-YYBJNFB	LOND-YYBETN4	LOND-YYBJWNI
LOND-YYBE67S	LOND-YYBEOY9	LOND-YYBE9NC	LOND-YYBJNOK	LOND-YYBEWMF	LOND-YYBJYD8
LOND-YYBE6DM	LOND-YYBEPR4	LOND-YYBE9UJ	LOND-YYBJNZH	LOND-YYBEWRU	LOND-YYBJZ9Z
LOND-YYBE6J9	LOND-YYBESDU	LOND-YYBE9X1	LOND-YYBJR1R	LOND-YYBEXEO	LOND-YYBE14E
LOND-YYBE6OO	LOND-YYBESZK	LOND-YYBEA8J	LOND-YYBJR51	LOND-YYBEZIP	LOND-YYBE4YI
LOND-YYBE6QI	LOND-YYBEXGW	LOND-YYBEAE8	LOND-YYBJRZ4	LOND-YYBEZKR	LOND-YYBE6C3
LOND-YYBE6XU	LOND-YYBEZ4D	LOND-YYBEAFP	LOND-YYBJY34	LOND-YYBJ898	LOND-YYBE7JY
LOND-YYBE6ZN	LOND-YYBEZFQ	LOND-YYBEAJF	LOND-YYBJY6O	LOND-YYBJ8I5	LOND-YYBEIXH
LOND-YYBE747	LOND-YYBEZP6	LOND-YYBEH8F	LOND-YYBJY89	LOND-YYBJBA5	LOND-YYBEJK1
LOND-YYBE76I	LOND-YYBJBMS	LOND-YYBEHED	LOND-YYBJY9Q	LOND-YYBJBGY	LOND-YYBEKAW

LOND-YYBE78R	LOND-YYBJBQO	LOND-YYBEHG8	LOND-YYBJYAH	LOND-YYBJBTJ	LOND-YYBEOEX
LOND-YYBE7BO	LOND-YYBJY1E	LOND-YYBEHQZ	LOND-YYBJYJ5	LOND-YYBJCYB	LOND-YYBEQYB
LOND-YYBE7H3	LOND-YYBJY4A	LOND-YYBEHTO	LOND-YYBJYK3	LOND-YYBJD1F	LOND-YYBET13
LOND-YYBE7K6	LOND-YYBJYBM	LOND-YYBEHYU	LOND-YYBJYNJ	LOND-YYBJDNG	LOND-YYBETG1
LOND-YYBE7RK	LOND-YYBJYHW	LOND-YYBEO4K	LOND-YYBE34B	LOND-YYBJDZ5	LOND-YYBEWEM
LOND-YYBEWO4	LOND-YYBJC6R	LOND-YYBJCJX	LOND-YYBJCXD	LOND-YYBJG7C	LOND-YYBJKSS
LOND-YYBJ8U9	LOND-YYBJCAO	LOND-YYBJCKP	LOND-YYBJE7K	LOND-YYBJGBF	LOND-YYBJKW4
LOND-YYBJB6X	LOND-YYBJCFZ	LOND-YYBJCN7	LOND-YYBJFAZ	LOND-YYBJKEU	LOND-YYBJM5M
LOND-YYBJBOM	LOND-YYBJCHE	LOND-YYBJCP8	LOND-YYBJG3W	LOND-YYBJKF3	LOND-YYBJMAT
LOND-YYBJRUN	LOND-YYBJRG7	LOND-YYBJR9K	LOND-YYBJQEX	LOND-YYBJPD4	LOND-YYBJNDF
LOND-YYBJTQY	LOND-YYBJYCI				

PANORAMIC Trial Collaborative Group:

Haroon Ahmed, Tanveer Ahmed, Damien Allcock, Sarah Barrett, Clare Bateman, Adrian Beltran-Martinez, Oluseye E Benedict, Magdalena Benysek, Nicholas S Berry, Nigel Bird, Laura Brennan, Gerard Burns, Daniel Butler, Mike Butler, Andrew Carson-Stevens, Jem Chalk, Zelda Cheng, Maria Coates, Lucy Cureton, Ruth Danson, Jennifer C Davies, Nigel de Kare-Silver, Michelle A Detry, Devesh Dhasmana, Jon Dickson, Melissa Dobson, Sarah Dowsell, Serge Engamba, Stacey Fisher, Robin Fox, Mark Fitzgerald, Eve Frost, Ushma Galal, Richard Gaunt, Sarit Ghosh, Ishtiaq Gilkar, Anna Goodman, Steve Granier, Radhika Gulati, Aleksandra Howell, Iqbal Hussain, Elizabeth Hadley, Victoria Harris, Jane Holmes, Simon Hutchinson, Marie Imlach, Greg Irving, Nicholas Jacobsen, James Kennard, Umar Khan, Kyle Knox, Christopher Krasucki, Layla Lavallee, Tom Law, Rem Lee, Nicola Lester, David Lewis, James Lunn, Claire I Mackintosh, Tracie-Ann Madden, Mehul Mathukia, Joe Marion, Micheal McKenna, Patrick Moore, Sam Mort, Seb Morton, Daniel Murphy, Lazaro Mwandigha, Rhiannon Nally, Chinonso Ndukauba, Emma Ogburn, Olufunto Ogundapo, Henry Okeke, Alice Packham, Amit Patel, Kavil Patel, Mahendra G Patel, Ruth Penfold, May Ee Png, Stavros Petrou, Satveer Poonian, Olajide Popoola, Alexander Pora, Vibhore Prasad, Rishabh Prasad, Ivy Raymundo-Wood, Omair Razzaq, Scot Richardson, Simon Royal, Heather Rutter, Afsana Safa, Christina T Saunders, Benjamin R Saville, Satash Sehdev, Tamsin Sevenoaks, Aadil Sheikh, Vanessa Short, Baljinder S Sidhu, Ivor Singh, Yusuf Soni, Andrew Ustianowski, Pete Wilson, David Wingfield, Michael Wong, Nick Wooding, Sharon Woods, Joanna Yong.
